# Transcriptomic analysis of *poco1*, a mitochondrial pentatricopeptide repeat protein mutant in *Arabidopsis thaliana*

**DOI:** 10.1186/s12870-020-02418-z

**Published:** 2020-05-12

**Authors:** Hossein Emami, Abhishek Kumar, Frank Kempken

**Affiliations:** 1 Department of Botany, Christian-Albrechts-University, Olshausenstr. 40, 24098 Kiel, Germany; 2 Present address: Institute of Bioinformatics, International Technology Park, Bangalore, 560066 India; 3 Present address: Manipal Academy of Higher Education (MAHE), Manipal, Karnataka 576104 India

**Keywords:** PPR protein, POCO1, Flowering time, Mitochondria, ABA signaling, RNA-seq, *A. thaliana*

## Abstract

**Background:**

Flowering is a crucial stage during plant development. Plants may respond to unfavorable conditions by accelerating reproductive processes like flowering. In a recent study, we showed that PRECOCIOUS1 (POCO1) is a mitochondrial pentatricopeptide repeat (PPR) protein involved in flowering time and abscisic acid (ABA) signaling in *Arabidopsis thaliana*. Here, we use RNA-seq data to investigate global gene expression alteration in the *poco1* mutant.

**Results:**

RNA-seq analysis was performed during different developmental stages for wild-type and *poco1* plants. The most profound differences in gene expression were found when wild-type and *poco1* plants of the same developmental stage were compared. Coverage analysis confirmed the T-DNA insertion in *POCO1*, which was concomitant with truncated transcripts. Many biological processes were found to be enriched. Several flowering-related genes such as *FLOWERING LOCUS T* (*FT*), which may be involved in the early-flowering phenotype of *poco1*, were differentially regulated. Numerous ABA-associated genes, including the core components of ABA signaling such as ABA receptors, protein phosphatases, protein kinases, and ABA-responsive element (ABRE) binding proteins (AREBs)/ABRE-binding factors (ABFs) as well as important genes for stomatal function, were mostly down-regulated in *poco1*. Drought and oxidative stress-related genes, including ABA-induced stress genes, were differentially regulated. RNA-seq analysis also uncovered differentially regulated genes encoding various classes of transcription factors and genes involved in cellular signaling. Furthermore, the expression of stress-associated nuclear genes encoding mitochondrial proteins (NGEMPs) was found to be altered in *poco1*. Redox-related genes were affected, suggesting that the redox state in *poco1* might be altered.

**Conclusion:**

The identification of various enriched biological processes indicates that complex regulatory mechanisms underlie *poco1* development. Differentially regulated genes associated with flowering may contribute to the early-flowering phenotype of *poco1*. Our data suggest the involvement of POCO1 in the early ABA signaling process. The down-regulation of many ABA-related genes suggests an association of *poco1* mutation with the ABA signaling deficiency. This condition further affects the expression of many stress-related, especially drought-associated genes in *poco1*, consistent with the drought sensitivity of *poco1*. *poco1* mutation also affects the expression of genes associated with the cellular regulation, redox, and mitochondrial perturbation.

## Background

One of the main interests in plant biology research is to find out how plant organelles are involved in plant growth, development and adaptation to stresses. Flowering is an intricate development stage, which is controlled by various genes from different pathways. Plants integrate a number of different signals to switch to flowering [1]. Alteration in the flowering time under various stresses or adverse conditions is a strategy taken by plants to ensure reproductive life [2]. The cooperative action of various genes that contribute to flowering gives rise to the onset of this process. Comprehensive knowledge of flowering time requires the identification of all factors involved in this process.

Abscisic acid (ABA) is an endogenous phytohormone that regulates the defensive responses of plants against biotic and abiotic stresses [3] and mediates vital processes of plant growth and development [2, 4, 5]. ABA biosynthesis and signaling are rapidly activated to respond to stresses and regulate stress-related genes required for plant tolerance. Hence, ABA is considered to be a major stress regulator [6]. Despite the widespread ABA synthesis among algal species, ABA-dependent responses could not be found, which suggest that the ABA-mediated signaling is a key evolutionary factor in the land plants to survive desiccation [7]. Also, important roles for ABA in other developmental processes have been described such as modulation of root patterning, root cell maintenance and root xylem formation [8–10]. Pyrabactin resistance 1 (PYR1)/pyr1-like (PYL) or regulatory components of ABA receptor (RCAR) proteins are intracellular receptors of ABA, either in the cytosol or the nucleus, which form a complex with the negative regulators of ABA signaling, protein phosphatase 2Cs (PP2Cs). As a result, phosphatases are inactivated and permit the interaction of sucrose nonfermenting 1-related protein kinases 2 (SnRK2s) with nuclear targets such as ABA-responsive element (ABRE) binding proteins (AREBs)/ABRE-binding factors (ABFs) to activate ABA-responsive gene expression [6, 11]. Mutants, which show alterations in ABA biosynthesis, perception, signaling, and response, show altered sensitivity to various stresses [12]. Several genes involved in stress responses function via ABA-independent and/or ABA-dependent signal-transduction cascades [4, 5]. Many ABA-inducible genes contain a conserved *cis*-acting ABRE, which can be recognized by AREB/ABFs [13, 14]. ABREs and AREB/ABFs are prerequisites for ABA-dependent gene expression [15].

Apart from the primary regulation of stress responses, ABA has a key role in flowering time. Through studies on ABA-insensitive mutants, which show an early-flowering phenotype and also exhibit inhibition of flowering by ABA treatment, the hindering effect of ABA on floral transition was demonstrated [15, 16]. The inhibitory effect of ABA on flowering time is mediated by ABSCISIC ACID INSENSITIVE 5 (ABI5) and other ABFs (ABF1, ABF3, and ABF4), by which *FLOWERING LOCUS C* (*FLC*) expression is promoted and subsequently floral integrators such as *FLOWERING LOCUS* T (*FT*) are repressed [17–19]. Studies found that flowering through *FT*, on the other hand, can be linked to stress-induced flowering to escape stress conditions [2].

Mitochondria are important with regards to sensing and integrating signals, stress responses and plant development [20]. Reproductive development is severely sensitive to mitochondrial mutations, which affect mitochondrial functions [21, 22]. However, molecular and genetic mechanisms behind mitochondrial activity and regulation during plant development are still mostly uncharacterized. In the case of any change in metabolic functions due to nonoptimal conditions, the communication between mitochondria and the nucleus will be altered to adapt to the new conditions. In mitochondria, reactive oxygen species (ROS) are produced as part of the normal metabolism of the mitochondrial electron transport chain (mETC). If the normal level is exceeded upon the perturbation of respiratory complexes, ROS leads to the alteration of the redox state and gene expression [23–25]. Retrograde signals regulate the expression of a large number of nuclear genes, among which are stress-responsive nuclear genes encoding mitochondrial proteins (NGEMPs) [20]. Notably, the phytohormone ABA significantly regulates mitochondrial function and can change the abundance of mitochondrial proteins [26]. It is therefore of interest to determine the molecular links between the mitochondrial function and regulation of nuclear genes, which most probably happen through retrograde signals.

In a recent study, we showed that a T-DNA insertional mutation in a mitochondrial PPR protein, POCO1, led to an early floral transition [27]. PPR proteins comprise a large family in land plants with 450 distinct members in *A. thaliana* and are involved in the post-transcriptional gene expression such as translation, splicing, editing, and stability of transcripts in organelles [28, 29]. *poco1* prevents proper mitochondrial function demonstrated by a lower rate of respiration, a low ATP level, and a higher generation of ROS. Additionally, multiple RNA editing defects were identified in *poco1*. *poco1* plants have decreased expression levels of *ABI5* and *FLC* and enhanced expression of *FT*. This could explain ABA insensitivity and the early-flowering phenotype of *poco1* plants. These plants also showed a higher susceptibility to drought stress.

In this study, we used RNA-seq to identify target genes contributing to the function of POCO1. Several flowering-associated genes, which may explain the acceleration of floral initiation in *poco1* were identified. In the *poco1* mutant, numerous genes related to ABA signaling and response, including ABA-induced stress genes, were down-regulated. Likewise, genes related to drought and oxidative stresses, redox-related genes, and mitochondrial perturbation marker genes were found to be differentially regulated. Genes associated with the cellular regulation and signaling were also found to be differentially regulated.

## Results

### Analysis of differentially expressed genes

**Fig. 1:**
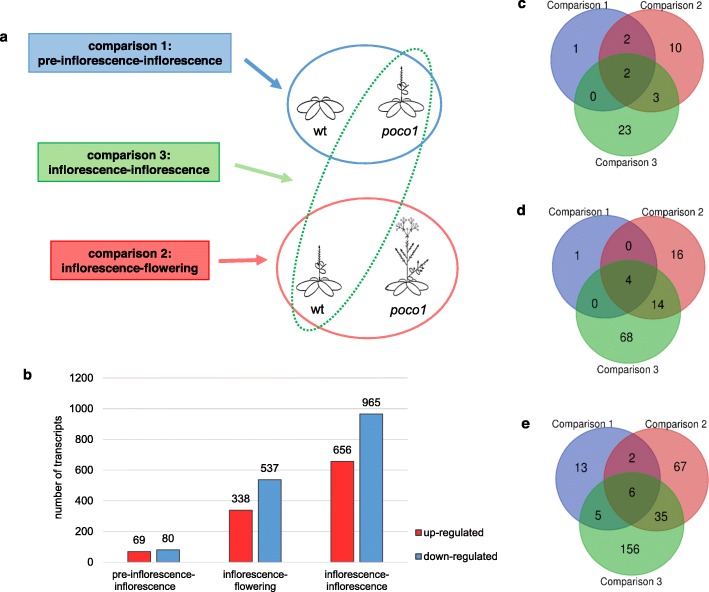
Experimental setup for RNA-seq and analysis of differentially expressed genes in three comparisons. Overview of the strategy for RNA-seq and analysis of differentially expressed genes. **a** RNAs were isolated at two time points. Stage 1: Wild-type plants without inflorescence stem and poco1 plants with inflorescence stem (pre-inflorescence-inflorescence). Stage 2: Wild-type plants with inflorescence stem and poco1 plants with flowers (inflorescence-flowering). Three RNA-seq comparisons were performed between wild-type and poco1. Comparisons 1 and 2 are referred to pre-inflorescence-inflorescence and inflorescence-flowering respectively. Comparison 3 is the comparison between wild-type and poco1 plants of the same developmental stage, in which both have inflorescence stem (inflorescence-inflorescence). **b** The number of up- and down-regulated differentially expressed genes between three comparisons. Differentially expressed genes were defined as those with a fold change either ≥ 2 or ≤ − 2 and an FDR < 0.05. The highest number of differentially expressed genes was observed for inflorescence-inflorescence. Venn diagrams showing unique or common differentially expressed genes in each gene of interest category as **c** Flowering-related genes, **d** ABA-related genes, and **e** Drought and oxidative stress-related genes. Venn diagrams were made by an online tool (http://bioinformatics.psb.ugent.be/webtools/Venn/)

The RNA samples in this study were isolated at two time points: The first time point was 20 days after sowing, when wild-type plants did not yet form any inflorescence stem but *poco1* plants already had (comparison 1: pre-inflorescence-inflorescence). The second time point was on 25 days after sowing, when wild-type plants had developed an inflorescence stem, and *poco1* plants flowered (comparison 2: inflorescence-flowering). One additional comparison was performed, in which both wild-type and *poco1* plants had developed an inflorescence stem (comparison 3: inflorescence-inflorescence). Thus, we analyzed wild-type and *poco1* plants of the same developmental stage (Fig. 1a). Isolated RNA samples were sequenced using the Illumina platform, and after quality analysis, the reads were further trimmed. Subsequently, RNA-seq analysis was performed. RNA-seq data have been deposited in the ArrayExpress database at EMBL-EBI (www.ebi.ac.uk/arrayexpress) under accession number E-MTAB-8912 (http://www.ebi.ac.uk/arrayexpress/experiments/E-MTAB-8912/). The total number of reads for each sample has been shown in Table 1. A total number of 2645 differentially expressed genes was identified.

**Table 1:** Number of reads of RNA-seq data. (The table belongs to the end of the first part of the result section “Analysis of the differentially expressed genes” first paragraph)

Samples	Genotypes	Developmental stage	Number of reads
HE954_1	wild-type	pre-inflorescence	138.218.582
HE954_2	wild-type	pre-inflorescence	92.869.772
HE954_3	wild-type	pre-inflorescence	77.430.678
HE954_4	*poco1*	inflorescence	81.985.354
HE954_5	*poco1*	inflorescence	105.508.752
HE954_6	*poco1*	inflorescence	87.081.482
HE958_1	wild-type	inflorescence	79.359.440
HE958_2	wild-type	inflorescence	88.105.132
HE958_3	wild-type	inflorescence	90.026.460
HE958_4	*poco1*	flowering	111.219.260
HE958_5	*poco1*	flowering	72.891.436
HE958_6	*poco1*	flowering	89.512.678

Gene expression alterations in *poco1* were studied in different developmental stages. As presented in Fig. 1b, pre-inflorescence-inflorescence showed the fewest number of differentially regulated genes. On the other hand, inflorescence-inflorescence, representing wild-type and *poco1* plants of the same developmental stage, showed the highest number of differentially expressed genes. All differentially expressed genes (fold changes either ≥2 or ≤ − 2, FDR < 0.05) allocated to the three comparisons are represented in (Additional file 1: Tables S1, S2, and S3).

To further analyze the genetic basis for *poco1* phenotypes such as early flowering and ABA insensitivity, genes associated with these categories were identified and their gene expression changes studied. Due to the drought stress susceptibility and an elevated level of ROS in *poco1*, which is highly linked to oxidative stress, genes related to these categories in all three comparisons were identified. Venn diagrams depicted the number of common and unique up- and down-regulated genes in each category (Fig. 1c, d, and e). To obtain more evidence of other possible effects in *poco1*, the expression profiles of genes associated with cellular regulation were studied. Differentially expressed genes related to the redox state, stomatal function, and mitochondrial perturbation were also identified.

**Fig. 2:**
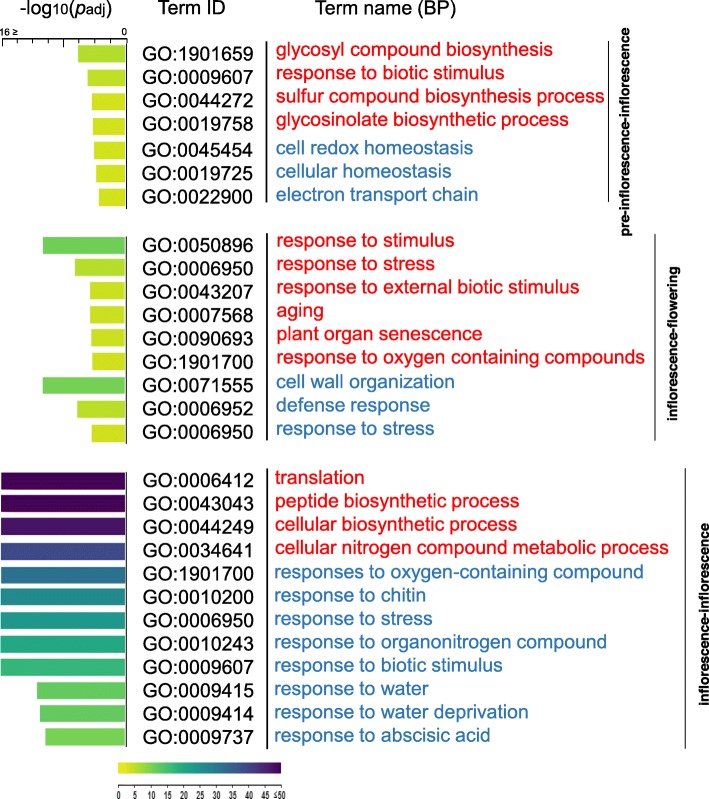
A summary of the GO enrichment analysis. A summary of the GO analysis in each comparison is represented. The adjusted p-values (Padj) are shown in negative log10 scale (capped at -log10 (Padj) ≤ 16). Biological processes terms in red and blue refer to the up- and down-regulated genes respectively. BP- Biological process

To understand the biological significance of gene expression in *poco1*, a gene ontology (GO) enrichment analysis was performed with the detected genes (fold changes either ≥2 or ≤ − 2, FDR < 0.05) in each comparison (Additional file 2: Figure S1). GO analysis revealed the important roles of enriched groups in the regulation of *poco1* in each comparison. Biological process GO terms related to biotic stresses and defense response such as “glycosyl compound biosynthesis,” “response to biotic stimulus,” “glycosinolate biosynthetic,” and “sulfur compound biosynthesis process” were over-represented in the up-regulated genes of pre-inflorescence-inflorescence (69 genes) (Additional file 2: Figure S1). The biological process GO terms “cell redox homeostasis,” “cellular homeostasis,” and “electron transport chain” were enriched in the down-regulated genes of pre-inflorescence-inflorescence (80 genes) (Additional file 2: Figure S1), which may be related to the higher generation of ROS in *poco1* [27]. GO enrichment of the up- and down-regulated genes of inflorescence-flowering (338 and 537 genes respectively) (Additional file 2: Figure S1) showed that various stress response-related processes are highly over-represented, which may indicate that different stresses allocate some identical pathways. Also in inflorescence-flowering, GO terms related to ageing and cell wall organization were enriched in up- and down-regulated genes respectively. GO enrichment of the up-regulated genes of inflorescence-inflorescence (656 genes) (Additional file 2: Figure S1) indicated that terms associated with “translation” and “peptide biosynthetic process,” as well as GO terms related to the biosynthetic and metabolic processes, were enriched. Biological process GO terms associated with nitrogen compound biosynthesis are over-represented. GO terms mainly related to stresses such as “responses to oxygen-containing compound,” “response to chemicals,” “response to chitin,” “response to stress,” “response to biotic stimulus,” “defense response to other organisms,” “response to organonitrogen compound,” “response to water deprivation,” and “response to abscisic acid” were enriched in the down-regulated genes of inflorescence-inflorescence (965 genes). The regulatory and functional attributions of drought stress responses, which are “responses to water deprivation” and “response to water,” were also among the enriched biological processes (Additional file 2: Figure S1). A summary of GO enrichment analysis is represented in Fig. 2.

Based on the 25 topmost up- and down-regulated genes (Additional file 1: Tables S1, S2 and S3) three genes are commonly up-regulated in all three comparisons: *PHOSPHATIDYLINOSITOL 4-KINASE GAMMA-LIKE PROTEIN* (*ATPI4Kɣ3*), *Cwf18* pre-mRNA splicing factor, and *PR5-LIKE RECEPTOR KINASE* (*PR5K*). Four genes were commonly down-regulated in all three comparisons: *TGG2*, leucine-rich repeat [LRR] family (*AT4G16880*), hypothetical protein (*AT5G22608*), and disease resistance protein family (*AT5G43740*). The up-regulation of *ATPI4Kɣ3* and the down-regulation of *TGG2* were demonstrated to lead to an ABA-insensitive phenotype [30, 31]. *Cwf18* pre*-*mRNA splicing factor was previously suggested to have a critical role in gene expression and abiotic stresses [32].

### Coverage analysis confirmed *poco1* T-DNA insertional mutation with truncated transcripts

**Fig. 3:**
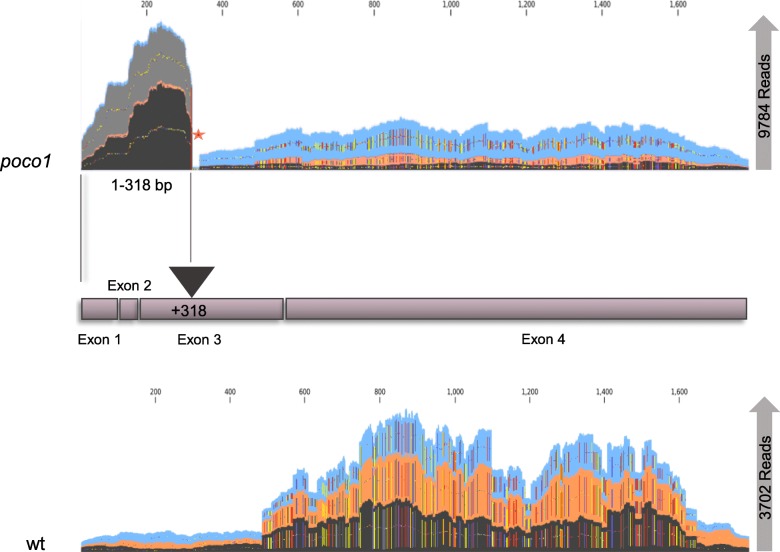
Analysis of *POCO1* transcript in wild-type and mutant. Visual representation of POCO1 reads in RNA samples of wild-type and poco1 mutants. The “Map reads to contigs” tool from the CLC Genomics Workbench 7.5.1 program was used. The total number of reads has been written on the right side for each genotype. poco1 has a T-DNA insertion at position + 318. Transcripts of poco1 show the truncated form and are highly abundant from the + 1 to + 318 position. A red star in poco1 mutants represents the gap in POCO1 sequence, which did not map to any transcript

A recent study showed that *poco1* carries a T-DNA insertion and was identified to be a homozygous T-DNA insertion mutant [27]. No *POCO1* transcript could be identified in *poco1* by RT-PCR. However, RNA-seq analysis from wild-type and *poco1* showed that *POCO1* (*AT1G15480*) is significantly up-regulated in *poco1* compared with wild-type. Therefore, we initially examined the transcript coverage in wild-type and *poco1*. Figure 3 shows that the abundance of the reads from + 1 to + 318 bp is extraordinarily high in *poco1*, which could not be observed for wild-type. Position + 318 is the position of the T-DNA insertion in the *POCO1* (FLAG_465F03). In the *poco1* mutant, a gap exists after position + 318, which did not map to any reads. This condition indicates the presence of truncated *POCO1* RNA in mutants, due to T-DNA insertion, which would not allow for translation of the POCO1 protein.

### Affected genes associated with flowering

Among differentially expressed genes in three comparisons, 41 genes related to flowering, including common flowering-related genes of widely known pathways, along with other genes that contribute to flowering were identified. Gene expression analysis of flowering-associated genes may help in unravelling the mechanism of floral transition in *poco1*. A heat map of flowering-associated genes displays the differential regulation of these genes in different comparisons (Additional file 3: Figure S2). Identified genes belong to the photoperiod and gibberellic acid pathways. Moreover, floral integrators and a photoreceptor associated with flowering, as well as an *FLC* specific regulator, are differentially regulated in *poco1* (Additional file 3: Figure S2). RNA-seq results identified flowering-associated genes, whose up- or down-regulation fits *poco1*’s phenotype. Based on the analysis of the differentially regulated genes associated with flowering, *poco1* leads to the alteration of gene expression that results in the acceleration of flowering. Some examples of identified differentially expressed genes in *poco1* associated with flowering are represented in Table 2.

**Table 2:** Some of differentially regulated genes in *poco1* and their involvement in flowering. (The table belongs to the end of the third part of the result section “Affected genes associated with flowering”)

Gene name	Gene regulation			Description	Reference
*ACR4*			↑	up-regulated during floral induction in the apical meristem	[33]
*Ankyrin*	↑	↑		up-regulated during floral induction in the apical meristem	[33]
*CRK6*		↓	↓	ROS sensing, signaling, mutants flower early	[34]
*CRK19*			↓	abiotic stress tolerance and hypersensitive response, mutants flower early	[34]
*DGR2*			↑	up-regulated during floral induction in the apical meristem	[33]
*ELF4-L1*			↓	circadian clock/photoperiod regulation of flowering, mutants flower early	[35]
*FLN1*			↑	up-regulated during floral induction in the apical meristem	[33]
*FLP1*		↑		floral induction, flowering time control, over-expression line flowers early	[36]
*FRL*			↓	induction of *FLC*	[37–40]
*FT*		↑		positive regulation of floral induction/flower development, mutants flower late	[41]
*GA3ox1*			↑	gibberellic acid biosynthetic pathway	[42, 43]
*GI*		↑		induction of flowering via the circadian clock/photoperiod, mutants flower late	[44]
*GID1B*		↑		gibberellic acid signaling pathway	[45]
*GRP7*		↑		regulation of flowering time, mutants flower late and over-expression line flowers early	[46]
*PHYE*			↓	repressor of flowering, phototransduction, mutants flower early	[47, 48]
*ROXY2*			↑	anther development	[49]
*TRM112A*		↑		up-regulated during floral induction in the apical meristem	[33]
*AT1G09390*			↑	up-regulated during floral induction in the apical meristem	[33]
*AT5G56120*			↑	up-regulated during floral induction in the apical meristem	[33]

Gene regulation symbols from left to right refer to the regulation in pre-inflorescence-inflorescence, inflorescence-flowering and inflorescence-inflorescence respectively. Gene regulation symbols- ↑: up-regulation; ↓: down-regulation

### *poco1* inactivates ABA signaling and response

A recent study revealed that *poco1* plants showed an insensitive phenotype to ABA regarding the primary root growth and flowering time. The study showed that *ABI5*, which acts at the core of ABA signaling, is down-regulated in *poco1* plants [27]. To enhance our understanding, the focus was laid on other factors associated with ABA signaling and response were searched in the differentially expressed genes in the RNA-seq results. A total of 104 genes, which are associated with ABA signaling and response in all three comparisons, were identified to be differentially expressed in *poco1*. A heat map of differentially expressed ABA-associated genes has been developed (Additional file 4: Figure S3). The majority of ABA-related differentially expressed genes were found in inflorescence-inflorescence and found mostly down-regulated. Interestingly, several genes functioning in the core of ABA perception and signaling were identified. *PYRABACTIN RESISTANCE 1-LIKE 5*/*REGULATORY COMPONENT OF ABA RECEPTOR 8* (*PYL5*/*RCAR8*) and *PYL9/RCAR1*, are involved in early ABA perception and signaling. PP2Cs such as *ABA*-*INSENSITIVE 1* (*ABI1*), (*ABI2*), *HYPERSENSITIVE GERMINATION 1* (*AHG3*), *HOMOLOGY TO ABI 1* (*HAB1*), and *HAB2* are also down-regulated in *poco1*. *ABI1* and *ABI2* are type 2C protein phosphatases and function in ABA signal transduction. *abi1* and *abi2* have an ABA-insensitive phenotype and prevent ABA signal transduction [50]. Protein kinases (SnRK2), *SNF1-RELATED PROTEIN KINASE 2.1* (*SnRK2.1*), and *SnRK2.8* were found to be down-regulated in *poco1*. *SnRK2.1* and *SnRK2.8* switch on ABA signaling by phosphorylation of different target proteins [51]. ABA-activated transcription factors, *ABRE-BINDING FACTOR 3* (*ABF3*), and *ABF4* are involved in ABA signal transduction. The involvement of *ABF3* and *ABF4* in water-deprivation response has been demonstrated [52, 53]. Calcium-dependent protein kinases (CDPKs) are Ca^2+^ binding sensory proteins and have been previously reported to be involved in ABA/stress signaling in *Arabidopsis* and other species [54, 55]. *CALCIUM*-*DEPENDENT PROTEIN KINASE 32* (*CPK32*) belongs to the ABA signaling component that regulates ABA-responsive gene expression via ABF4 phosphorylation. Some examples of differentially expressed genes in *poco1* that were identified to be associated with ABA signaling and response are represented in Table 3.

**Table 3:** Some of the differentially regulated genes in *poco1* associated with ABA. (The table belongs to the end of the fourth part of the result section “*poco1* inactivates ABA signaling and response”)

Gene name	Gene regulation			Description	Reference
*ABF3*			↓	ABA signaling, ABA and water deprivation response, mutants show ABA-insensitivity	[52]
*ABF4*			↓	ABA signaling, ABA and water deprivation response, mutants show ABA-insensitivity	[52]
*ABI1*			↓	negative regulator of ABA signaling, mutants show ABA insensitivity	[56]
*ABI2*			↓	negative regulator of ABA signaling, mutants show ABA insensitivity	[56]
*AHG3*			↓	negative regulator of ABA signalling/ water deprivation	[57]
*AtPI4Kɣ3*	↑	↑	↑	response to ABA, over-expression line shows ABA insensitivity and reduced induction of *ABI5*	[30]
*CPK32*			↓	ABA signaling and response, over-expression line show ABA-hypersensitivity and enhanced expression of ABA-regulated genes	[58]
*CYP707A3*		↓		ABA catabolic and metabolic processes, involved in dehydration and rehydration	[59]
*HAB1*			↓	negative regulator of ABA signaling	[56]
*HAB2*			↓	negative regulator of ABA signaling	[56]
*LTP3*	↓	↓	↓	ABA response	[60]
*LTP4*	↓	↓	↓	ABA response	[61]
*MARD1*			↓	response to ABA	[62]
*MLP43*		↓	↓	positive regulator of ABA signaling, involved in drought tolerance	[63]
*NCED4*		↑	↓	ABA biosynthesis	[64]
*PYL5*/*RCAR8*	↓			ABA signaling and response, drought stress response	[65]
*PYL9/RCAR1*			↓	ABA signaling and response, mutants are ABA-insensitive	[65, 66]
*RAB18*			↓	ABA and abiotic stress-responsive	[63]
*RPK1*			↓	ABA signaling pathway, ABA and water deprivation response, altered stress-induced responses in mutants	[67, 68]
*SnRK2.1*			↓	ABA signaling, water deprivation response	[69]
*SnRK2.8*			↓	ABA signaling, water deprivation response, over-expression line enhances drought tolerance	[52]
*SYP121*		↓		response to ABA	[70]

Gene regulation symbols from left to right refer to the regulation in pre-inflorescence-inflorescence, inflorescence-flowering and inflorescence-inflorescence respectively. Gene regulation symbols- ↑: up-regulation; ↓: down-regulation

### Identification of genes associated with drought and oxidative stresses

Our recent study showed that *poco1* plants are more sensitive to drought stress and accumulate a higher amount of ROS [27]. Therefore, attention was focused on identifying differentially expressed genes associated with drought and oxidative stresses were searched from differentially expressed genes in the RNA-seq results. In this category, a relatively high number of genes were identified (Additional file 5: Figure S4). In addition to their functions in ABA signaling and response, many of ABA-induced genes act in stresses, especially drought stress. Many stress genes, which are highly induced by ABA, such as *LIPID TRANSFER PROTEIN 3* (*LTPs*), *ERDs*, *RESPONSIVE TO DESICCATION (RDs)*, *COLD-REGULATED 47* (*COR47*)*, COLD-REGULATED 413-PLASMA MEMBRANE 2* (*COR413-PM2*), and *RAB18*, are repressed in *poco1* (Additional file 5: Figure S4). Moreover, genes that are regulators of ABA signaling such as *PYL5*/*RCAR8*, *PYL9*/*RCAR1*, *ABI1*, *ABI2*, *SnRK2.1*, *SnRK2.8*, *ABF3*, and *ABF4* were reported to function in ABA-mediated responses to abiotic stresses [71, 72]. The expression level of authentic drought-induced genes such as *RAB18* [73] and *RD29A* [74], which are marker genes of ABA signaling, is down-regulated in *poco1*. Overall, these results may ideally explain the higher sensitivity of *poco1* to drought stress compared to wild-type plants.

In *poco1* a higher amount of ROS was detected than in wild-type [27]. A higher level of ROS is linked to oxidative stress conditions. Many oxidative stress-related genes in *poco1* were found to be differentially regulated compared with wild-type plants (Additional file 5: Figure S4). Many of the identified genes were found to have an oxidoreductase activity, which may be associated with the higher accumulation of ROS in *poco1*. Some examples of the identified differentially expressed genes in *poco1* associated with drought and oxidative stresses are represented in Table 4.

**Table 4:** Some of the differentially regulated drought and oxidative stress genes in *poco1*. (The table belongs to the end of the fifth part of the result section “identification of genes associated with drought and oxidative response”)

Gene name	Gene regulation			Description	Reference
*COR47*			↓	response to water deprivation	[75]
*COR413-PM2*			↓	cellular response to water deprivation	[76]
*ERD1*			↓	drought stress tolerance	[77]
*ERD10*			↓	response to water deprivation	[78]
*LTP3*	↓	↓	↓	response to water deprivation	[79]
*LTP4*	↓	↓	↓	response to water deprivation	[79]
*FRO4*		↑		oxidation reduction process	[80]
*FRO7*			↓	oxidation reduction process	[80]
*LTI78/RD29A*				Response to water deprivation, response to ROS	[81]
*PRXQ*		↓	↑	cell redox homeostasis, cellular response to oxidative stress	[82]
*PRXR1*			↓	response to oxidative stress	[83]
*RD28*			↑	response to desiccation	[84]

Gene regulation symbols from left to right refer to the regulation in pre-inflorescence-inflorescence, inflorescence-flowering and inflorescence-inflorescence respectively. Gene regulation symbols- ↑: up-regulation; ↓: down-regulation

### Identification of genes associated with cellular regulation and signaling

To provide an insight into the regulatory network that controls *poco1*’s cellular metabolism, different classes of transcription factors and genes involved in cellular signaling were identified from differentially expressed genes. Numerous transcription factors have been identified to be differentially expressed in *poco1* in all three comparisons, which are classified to bHLH, bZIP, CCCH zinc finger, C2H2 zinc finger, CO-like, ERF, GATA, GRAS, HMG, Homeobox, HSF, mTERF, MYB, MYB-like, NAC, NF-Y, PLATZ, RWP-RK, RAV, Sigma 70-like, TCP, and WRKY transcription factors (Additional file 6: Figure S5). Similar to other analyses, pre-inflorescence-inflorescence and inflorescence-inflorescence have the lowest and the highest number of regulated genes encoding transcription factors respectively. The majority of differentially regulated transcription factors in inflorescence-flowering were up-regulated. Conversely, the majority of differentially regulated transcription factors in inflorescence-inflorescence were down-regulated. Differentially regulated genes from bHLH, MYB-like, and NAC transcription factor family showed up-regulation in inflorescence-flowering. Conversely, MYB-like and NAC transcription factors showed down-regulation in inflorescence-inflorescence. This is also the case for the majority of genes encoding Homeobox and MYB transcription factors. The highest number of regulated genes encoding transcription factors belongs to the ERF transcription factor family. Some examples of differentially expressed genes encoding transcription factors in *poco1* are represented in Table 5.

**Table 5:** List of some of the differentially regulated genes encoding transcription factors in *poco1*. (The table belongs to the end of the sixth part of the result section “identification of genes associated with cellular regulation and signaling”)

Gene name	Gene regulation			TF	Description	Reference
*FBH2*	↑	↑		bHLH	photoperiod-independent effect on flowering, over-expression line with an early-flowering phenotype	[85]
*PRE1*			↑	bHLH	over-expression line with an early-flowering phenotype, gibberellic acid-dependent response	[86]
*MYC2*			↓	bHLH	positive regulator of ABA signaling	[87]
*MYB2*		↑		MYB	response to ABA, response to water deprivation	[88]
*MYB20*		↑		MYB	positive regulator of ABA signaling	[89]
*MYB32*			↓	MYB	response to ABA	[90, 91]
*MYB51*			↓	MYB	response to ABA	[92]
*MYB73*			↓	MYB	interaction with ABA signaling components	[93]
*NAC089*			↓	NAC	negative regulation of flower development	[94]
*RAV1*			↓	ERF	negative regulation of flower development	[95]
*TEM1*			↓	RAV	*FT* repressor, mutants flower early, overexpression line flowers late	[95]
*WRKY15*		↓	↓	WRKY	early H2O2 responsive, over-expression line disrupts ROS and mitochondrial retrograde signaling	[96, 97]
*WRKY25*			↓	WRKY	response to various abiotic stresses, ABA response, over-expression line shows ABA sensitivity	[98–100]
*WRKY33*			↓	WRKY	response to various abiotic stresses, ABA response, over-expression line shows ABA sensitivity	[98–100]
*WRKY46*			↓	WRKY	regulation of ABA signaling and response to water deprivation	[101]

Gene regulation symbols from left to right refer to the regulation in pre-inflorescence-inflorescence, inflorescence-flowering and inflorescence-inflorescence respectively. Gene regulation symbols- ↑: up-regulation; ↓: down-regulation; TF- transcription factor

Studies have reported that ABA affects the induction of many genes encoding transcription factors [102]. As the most abundant class of transcription factors in plants, MYBs are involved in plant development, hormone signal transduction, and abiotic stress tolerance [88]. WRKY transcription factors are also one of the largest transcription factors, functioning in biotic and abiotic stresses [98, 103]. The expression of the WRKYs in *poco1* is mostly down-regulated (Additional file 6: Figure S5). WRKY2 was reported to act as a transcriptional regulator of AREBs/ABFs through binding W-box sequences (a core binding site for WRKYs) in the promoter regions of AREBs/ABFs [98]. ABA-responsive genes such as *ABF4*, *ABI5*, *MYB2*, and *RAB18* are target genes of WRKYs. Several genes involved in stress adaptation such as *RD29A* and *COR47* were reported to be downstream of *WRKYs* [98]. mTERFs are another group of transcription factors that are involved in organellar gene expression. They are mostly up-regulated in *poco1*. Collectively, these results suggest that the activity of a number of transcription factors that regulate critical biological processes may be altered in *poco1*.

Signaling molecules modulate diverse cellular responses and affect plant development, hormone and stress response pathways [68, 104]. Analysis of RNA-seq data showed that several genes encoding proteins associated with cellular signaling such as receptor-like kinases (RLKs), receptor-like proteins (RLPs), mitogen-activated protein kinases (MAPKs), and leucine-rich repeat protein kinases (LRR-RKs) are differentially regulated in *poco1* (Additional file 7: Figure S6).

### Genes associated with mitochondrial perturbation show an altered expression profile

The mitochondrion plays an important role in sensing stresses and directing the cellular response [20, 105]. Mitochondrial function is disturbed by stresses, and feedback mechanisms will be activated to regulate gene expression to sustain mitochondrial and cellular functions [105, 106]. Signals are transmitted from mitochondria to the nucleus (retrograde signal), leading to the corresponding responses by changing the nuclear gene expression. POCO1 is localized to mitochondria, and its loss of function led to mitochondrial dysfunction [27]. Therefore, the impact of retrograde signals on the expression of nuclear genes encoding mitochondrial proteins (NGEMPs) is plausible in *poco1*. Consistently, the RNA-seq analysis identified several NGEMPs that are targets of the mitochondrial perturbation status (Additional file 7: Figure S6). Thirty-seven genes associated with mitochondrial perturbation were identified to be differentially regulated in *poco1* in all three comparisons. A differential expression pattern of these genes hints to the existence of different pathways and signals in *poco1*, through which mitochondria communicate with the nucleus.

Transcripts encoding mitochondrial HSPs are particularly involved in mitochondrial dysfunctions as part of retrograde signals [107]. Two Mitochondrial *HSPs*, *HSP60*, and *mtHsc70–1* are found among the differentially expressed genes up-regulated in *poco1*. It was previously suggested that many of NGEMPs such as *HSP70*, *AOX1a*, and *BCS1* may be truly ABA-responsive, as their transcript abundance was changed after ABA treatment [108]. The newly characterized gene family, *domain of unknown function 295* (*DUF295*), was reported to be induced in *Arabidopsis* mutants with mitochondrial dysfunctions [109, 110]. A member of the *DUF295* gene family (*ATDOA11*) was found up-regulated in *poco1*. Some examples of the identified differentially expressed NGEMPs in *poco1* are represented in Table 6.

**Table 6:** Some of the differentially regulated NGEMPs in *poco1*. (The table belongs to the end of the seventh part of the result section “Genes associated with mitochondrial perturbation show an altered expression profile”)

Gene name	Gene regulation		Description	Reference
*AOX1a*		↓	mitochondria-nucleus signaling, alternative respiration	[111]
*AOX1d*	↑		mitochondria-nucleus signaling, alternative respiration	[111]
*ATDOA11*		↑	mitochondrial dysfunctions	[109]
*CRF6*	↑		mitochondrial retrograde response	[112]
*ERD5*	↑		mitochondria proline catabolic pathway, water deprivation response	[113]
*HSP60*		↑	protein import into mitochondrial intermembrane space, involved in mitochondrial dysfunctions as part of retrograde signals	[107]
*mtHsc70–1*		↑	response to unfolded proteins, involved in mitochondrial dysfunctions as part of retrograde signals	[107]

Gene regulation symbols from left to right refer to the regulation in inflorescence-flowering and inflorescence-inflorescence respectively. Gene regulation symbols- ↑: up-regulation; ↓: down-regulation

### Cellular redox state may be affected in *poco1*

The redox cascade of the mitochondrial electron transport chain generates redox signals, which can further partake in gene expression and regulation. Redox-based signaling may be a crucial constituent in mitochondria-nucleus communication [105, 114]. Increased ROS directly leads to the alteration of redox status [24]. Due to the increased ROS level, alteration in the cellular redox status in *poco1* is relevant. Many redox-related genes such as glutaredoxins (*GRXs*), glutathione s-transferases (*GSTs),* thioredoxins (*TRXs*), and rotamase cyclophilins (*ROCs*) were found among differentially expressed genes (Additional file 8: Figure S7). Oxidoreductases such as glutaredoxins (*GRXs*), which have peroxidase activity, are involved in different cellular processes, especially oxidative stresses [115]. Several members of the *GRX* gene family that are involved in the cell redox homeostasis such as *ROXY2*, *ROXY3*, *ROXY8*, *ROXY9*, *ROXY12*, *ROXY13*, *ROXY14*, *ROXY15*, *ROXY17*, *ROXY20*, and *ROXY21* are found to be differentially expressed in *poco1.* Several *GSTs* were found differentially regulated in *poco1*. Except one, all differentially regulated *GSTs* identified in inflorescence-inflorescence are down-regulated. However, regulated *GSTs* in inflorescence-flowering show a different expression pattern than inflorescence-inflorescence, in which four of the *GSTs* show up-regulation and three of them show down-regulation. These results support the hypothesis of cellular redox alteration in *poco1*, which may validate the role of POCO1 in mitochondrial function. Some examples of the identified differentially expressed genes in *poco1* associated with redox state are represented in Table 7.

**Table 7:** Some of the differentially regulated genes in *poco1* associated with the cellular redox state. (The table belongs to the end of the eighth part of the result section “cellular redox state may be affected in *poco1*”)

Gene name	Gene regulation			Description	Reference
*GSTU4*		↑	↓	degradation of H2O2, cellular redox homeostasis	[116]
*GSTF9*	↑			glutathione metabolic process	[117]
*GSTF12*		↑		glutathione metabolic process	[118]
*GSTU16*			↓	glutathione metabolic process	[116]
*ROC2*			↑	protein folding, connecting hormone signals to redox homeostasis in stresses	[119]
*ROC4*		↓	↑	protein folding, connecting hormone signals to redox homeostasis in stresses	[119]
*ROXY3*			↑	cell redox homeostasis	[120]
*ROXY8*			↓	cell redox homeostasis	[121]
*ROXY9*		↑	↓	cell redox homeostasis	[122]
*ROXY12*	↓		↑	cell redox homeostasis	[123]
*ROXY13*	↓		↑	cell redox homeostasis	[123]
*TRXz*			↑	cell redox homeostasis	[124]
*TRX5*			↓	cell redox homeostasis, oxidation-reduction process	[125]

Gene regulation symbols from left to right refer to the regulation in pre-inflorescence-inflorescence, inflorescence-flowering and inflorescence-inflorescence respectively. Gene regulation symbols- ↑: up-regulation; ↓: down-regulation

### Effect of *poco1* on stomatal function

One of the most important strategies of plants, which have evolved to adapt to adverse conditions, especially drought stress, is the control of stomatal aperture. ABA-mediated stress responses involve alterations in gene expression, which finally may affect the regulation of stomatal closure to regulate water loss. *poco1* plants are ABA-insensitive and susceptible to drought stress. Therefore, stomatal dysfunction in *poco1* is highly possible. RNA-seq results identified genes that play crucial roles in stomatal closure (Additional file 8: Figure S7). *GLUCOSIDE GLUCOHYDROLASE 2* (*TGG2*), a highly abundant myrosinase in guard cells, is strongly down-regulated in *poco1* in all three comparisons (lowest fold change in pre-inflorescence-inflorescence and inflorescence-flowering and the sixth lowest fold change in inflorescence-inflorescence) (Additional file 1: Table S1, S2 and S3). *TGG1* revealed a differential expression pattern in inflorescence-flowering and inflorescence-inflorescence. Cyclic nucleotide-gated channels (*CNGCs*), a family of plant ion channels, are expressed in response to abiotic stresses leading to the tolerance mechanism [126]. Another hint for potential stomatal failure comes from the down-regulation of *RESPIRATORY BURST OXIDASE HOMOLOGUE D* (*RBOHD*) in *poco1* [127]. The function of RBOHD was reported to be impaired in *gca2* ABA-insensitive mutant [128]. *GRP7* is expressed extensively in guard cells and influences stomatal opening and closure, thereby causes lowered dehydration tolerance [129, 130]. These results propose that *poco1* mutation may lead to stomatal failure. Some examples of the identified differentially expressed genes in *poco1* associated with stomatal function are represented in Table 8.

**Table 8:** Some of the differentially regulated genes in *poco1* associated with stomatal function. (The table belongs to the end of the ninth part of the result section “effect of *poco1* on stomatal function”)

Gene name	Gene regulation			Description	Reference
*ABI1*			↓	regulation of stomatal movement, mutants failed to activate anion channels in guard cells	[131, 132]
*ABI2*			↓	regulation of stomatal movement, mutants failed to activate anion channels in guard cells	[131, 132]
*CNGC1*			↓	highly expressed in guard cells, ion channel	[133]
*CNGC6*			↓	highly expressed in guard cells, ion channel	[133]
*GRP7*		↑		enhancement of stomatal opening	[130]
*MYB44*			↓	over-expression line shows enhanced stomatal closure	[91]
*RBOHD*			↓	increasing cytosolic ca^2+^, induced by ABA	[127]
*RPK1*			↓	mutants show insensitivity in ABA-induced stomatal closure	[134]
*TGG1*		↓	↑	regulation of stomatal movement, ABA-mediated stomatal closure	[135, 136]
*TGG2*	↓	↓	↓	regulation of stomatal movement, ABA-mediated stomatal closure	[135, 136]

Gene regulation symbols from left to right refer to the regulation in pre-inflorescence-inflorescence, inflorescence-flowering and inflorescence-inflorescence respectively. Gene regulation symbols- ↑: up-regulation; ↓: down-regulation

## Discussion

Transition to flowering is a critical step in the plant life cycle as it ensures the plant species continuity. Various factors involved in flowering have been identified. However, the characterization of POCO1, a mitochondrial PPR protein, whose corresponding mutant exhibited an early-flowering phenotype, would provide additional information regarding mitochondria-nucleus interactions. In this study, RNA-seq data were used to better understand POCO1’s function and to determine other factors that could lead to *poco1*’s phenotype. Interestingly, the majority of differentially expressed genes were identified through inflorescence-inflorescence, in which the plants are in the same developmental stage, which may suggest a high variation in metabolic processes in each same developmental stage between wild-type and *poco1* plants. Also, it may be due to the developmental reprogramming before sexual reproduction, which may have occurred more intensively in *poco1*. Besides, although being in the same developmental stage, they are not the same age. The coverage analysis of *POCO1* confirmed the truncated transcripts in *poco1* plants, which would not allow for the translation of functional POCO1.

The GO enrichment for the up- and down-regulated genes of each comparison suggests the crucial roles of those biological processes, in particular, the over-representation of many processes associated with response to stresses in regulating the developmental processes in *poco1*. Enriched biological processes related to the defense response may be due to the interaction of plant defense pathways and transition to the reproductive phase. A significant link between the regulation of glucosinolate content and flowering time in *Aethionema arabicum* (Brassicaceae) has been identified. FLC was determined to be the potential regulator of glucosinolate content [137]. Moreover, the glucosinolate and glycosinolate biosynthetic processes (enriched for the up-regulated genes in pre-inflorescence-inflorescence) are considered as principal regulators in the transition phase and facilitate the protection of plants against pathogens during flowering [138]. These compounds are composed of sulfur and nitrogen. Interestingly, *TGG2*, which is highly down-regulated in *poco1* in all comparisons, was demonstrated to be required for glucosinolate breakdown [135]. GO terms associated with ageing may emphasize the forefront developmental maturation of *poco1*, and as a logical consequence of ageing-related processes, cell wall organization and biogenesis were shown to be enriched for the down-regulated genes of inflorescence-flowering. The GO terms “translation” and “peptide biosynthetic process,” along with terms associated with “ribosome assembly,” which are highly enriched for the up-regulated genes of inflorescence-inflorescence, may indicate control of ribosomes. Hence, de novo protein synthesis is essential for the floral transition in *poco1*. Alteration in the translational machinery has been reported in *A. thaliana* under stress conditions [139]. This condition may highlight the importance of the translation apparatus in *poco1,* which bears with unfavorable conditions. Accordingly, *Cwf18* pre*-*mRNA splicing factor (among the top 10 most up-regulated genes in all three comparisons) was reported to function in the early response to abiotic stresses. *Cwf18* pre*-*mRNA splicing factor was suggested to function in the gene expression process and act along with proteins that function as part of the ribosome [32]. Therefore, its over-expression may further support the reprogramming of stress-induced transcriptional events in *poco1*. Studies have reported that plants can utilize the required nitrogen from organic compounds such as proteins and amides [140, 141]. In line with the GO biological process terms related to nitrogen compound biosynthesis processes, protein and amide biosynthesis processes are highly enriched for the up-regulated genes of inflorescence-inflorescence. Availability of nitrogen is a limiting factor for plant growth and development, which controls developmental phase change [142, 143]. Some studies showed that a higher nitrogen condition promoted flowering in *Arabidopsis* [144–147]. The GO terms “response to abscisic acid” and “response to water deprivation,” which are observed for the down-regulated genes of inflorescence-inflorescence, may explain the impaired ABA signaling and susceptibility of *poco1* to drought stress.

**Fig. 4:**
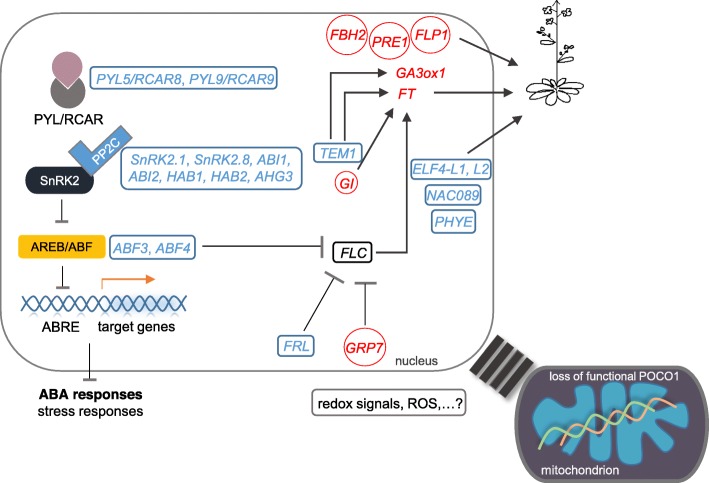
Affected genes in core components of ABA signaling and flowering in *poco1* in a schematic representation. In Arabidopsis, multiple factors affect flowering time to adapt the unfavorable conditions. In poco1 mutants, the core ABA signaling genes (PYRs/PYLs/RCARs, PP2Cs, SnRK2s and AREBs/ABFs) are down-regulated, which may lead to an impaired ABA and stress response. Down-regulation of ABF3 and ABF4, FRL and up-regulation of GRP7 may have an inhibitory effect on FLC expression. Besides, down-regulation of TEM1 and up-regulation of GI may induce FT expression. Down-regulation of TEM1 also induces the gibberellic acid biosynthesis gene, GA3ox1. Down-regulation of ELF4-L1, ELF4-L2, NAC089 and PHYE and up-regulation of FLP1, PRE1 and FBH2 are consistent with the early-flowering phenotype. Red and blue genes indicate up- and down-regulated genes respectively. Arrows and dashed arrows are indicative of inducing and inhibiting effects respectively

We found several genes functioning in promoting flowering and therefore are crucial to the early floral transition in *poco1*. Consistent with our recent study [27], the floral integrator *FT* is strongly up-regulated in *poco1* in inflorescence-flowering. Interestingly, one of its integrators, *GI*, was also found to be up-regulated in *poco1* in inflorescence-flowering. Expression of *GI* was previously reported to be a stress escape response, and the early floral transition in *Arabidopsis* in response to stress requires GI [148]. Except for GI, FT is also involved in stress-induced flowering [148, 149], which is an indication of the important role of these genes under unfavorable conditions to shorten the life cycle by promoting floral transition. Beyond that, these data strengthen the idea that the early flowering in *poco1* may be related to stress-induced flowering. This result is consistent with GO enrichment analysis of all differentially expressed genes, in which many terms were found related to response to stresses. From the RNA-seq results, we also identified that a repressor of *FT*, *TEMPRANILLO 1* (*TEM1*)*,* and a promoter of *FLC, FRIGIDA-LIKE* (*FRL*)*,* both showed down-regulation in *poco1*. Besides, loss of function of GRP7 was reported to increase the total functional sense *FLC* transcript and delays flowering time [150]. This result further highlights the hypothesis that the early flowering of *poco1* occurs via repression and elevation of *FLC* and *FT* respectively (Fig*.* 4). Except for the direct repression of *FT*, TEM1 also suppresses the expression of *GA3ox1* by directly binding to the *GA3ox1* loci [151]. A gibberellic acid biosynthesis gene *GA3ox1* was demonstrated to have a predominant role in plant development [42, 43]. Two functionally redundant gibberellin receptors, *GIBBERELLIN*-*INSENSITIVE DWARF 1* (*GID1B*) and *GID1C* [45] from the gibberellic acid signaling pathway, were differentially regulated in *poco1*. *PACLOBUTRAZOL RESISTANCE 1* (*PRE1*) over-expression line leads to the gibberellin-dependent response and activates a branch pathway of gibberellin signaling [86]. *FLOWERING PROMOTING FACTOR 1* (*FLP1*), was demonstrated to promote flowering time in the gibberellic acid-dependent signaling pathway in *Arabidopsis* [152]. These results may suggest a role for gibberellic acid in the floral transition of *poco1*. Besides, the up-regulation of *FBH2*, *PRE1*, and *FLP1* and the down-regulation of *PHYTOCHROME E* (*PHYE*), *EARLY FLOWERING*-*LIKE1* (*ELF4-L1*), *RELATED TO ABI3/VP1 1* (*RAV1)*, *NAC089, CYSTEINE-RICH RECEPTOR-LIKE KINASE 6* (*CRK6*), and *CRK19* allow an early-flowering phenotype (Fig*.*4).

Loss of function of POCO1 leads to the ABA-insensitivity phenotype [27]. We provided evidence that numerous genes associated with ABA signaling and response are down-regulated in *poco1*. *ATPI4Kɣ3* is the most up-regulated gene in *poco1* in pre-inflorescence-inflorescence and is also among the highest up-regulated genes in other two comparisons (Additional file 1: Table S1, S2 and S3). The function of ATPI4Kɣ3 is important during development and under abiotic stress conditions. Over-expression of *ATPI4Kɣ3* leads to the increased tolerance to ABA with reduced induction of *ABI5,* which is consistent with *poco1*. *RECEPTOR*-*LIKE PROTEIN KINASE 1* (*RPK1*) is involved in early ABA perception in *Arabidopsis* and acts as a regulator of ABA signaling in early steps. Therefore, it affects many downstream genes in ABA signal transduction [67, 68]. The RNA-seq analysis identified genes from the different components of ABA signaling: ABA receptors (*PYL5*/*RCAR8* and *PYL9/RCAR1*), protein phosphatases (*ABI1, ABI2, AHG3*, *HAB1*, and *HAB2*), protein kinases (*SnRK2.1* and *SnRK2.8*), and AREB/ABFs (*ABF3* and *ABF4*). Therefore, ABA signaling pathways are disrupted from the early perception to the expression of many ABA-responsive genes in *poco1* (Fig*.*4). Functional ABA signaling is essential for stress tolerance, particularly drought stress [4, 5, 11]. Many genes associated with drought stress contain *cis*-acting ABRE and dehydration-responsive element (DRE) [153]. Studies have demonstrated that ABFs bind to ABRE elements to activate ABA-responsive gene expression, which is crucial to drought stress tolerance [16, 72]. Therefore, the down-regulation of *ABFs* in *poco1* such as *ABF3* and *ABF4* is likely to be one possible scenario for the down-regulation of many ABA-induced stress-responsive genes, as shown in this study. Another intriguing feature of ABFs is their impact on floral transition. It has been reported that except ABI5, other ABFs can distinctly promote the expression of *FLC* via binding to ABRE elements in the promoter region of *FLC* [18]. Thus, it is probable that except ABI5, ABF3 and ABF4 have a direct effect on the repression of *FLC* in *poco1* (Fig*.*4)*.* Of particular note, the inhibitory role of ABFs on *FLC* expression is possibly adjusted through SnRK2s, which function to phosphorylate ABFs. This modulation directly affects floral transition [18]. However, *FLC* and *ABI5* were not identified as differentially expressed genes in the RNA-seq analysis. The up-regulated expression of *NCED4* in inflorescence-flowering and the down-regulated expression of *CYP707A3* in the same comparison may suggest an imbalance in ABA biosynthesis and catabolism in *poco1*. This imbalance can also be observed in inflorescence-inflorescence, where *NCED4* is down-regulated, but no genes with ABA catabolism function were detected.

Many well-known positive effectors or regulators of drought stress such as *RDs*, *ERDs*, *RAB18,* and *COR47* were found down-regulated in *poco1*. Consequently, as shown in our recent study, *poco1* plants were more susceptible to drought stress, and thus, *poco1* negatively regulates drought response. Although ABA initially demonstrated to orchestrate abiotic responses, further studies reported the additional involvement of ABA in abiotic stresses. ABA confers resistance to pathogens and diseases [154, 155]. Enriched biological processes associated with abiotic stresses thus may allegedly be due to the ABA signaling impairment in *poco1*, which may lead to the alteration in the expression of biotic stress-associated genes. Our RNA-seq analysis also identified many genes associated with the oxidation-reduction process, which may refer to the elevated intracellular levels of ROS, as observed previously in *poco1* plants [27].

An important role of ABA in drought stress is to modulate stomatal closure, preventing less transpirational water loss [156]. ABA promotes stomatal closure by regulating the many genes involved in dehydration tolerance [157]. Our transcriptomic data support the hypothesis that the stomatal closure in *poco1* may fail. *TGG1* and *TGG2* are two myrosinases and demonstrated to be an important components of the ABA signaling in guard cells [136]. It was reported that TGG1 and TGG2 have functional redundancy in ABA signaling in *Arabidopsis* guard cells [31]. Studies have demonstrated that SnRK2s have critical functions in stomatal movements [158]. Therefore, it can be speculated that the lower induction of *SnRK2.1* and *SnRK2.8* might have negative effects on stomatal regulation in *poco1*. Furthermore, alteration in the expression of *GRP7*, *RPK1*, *CNGCs*, *RBOHD*, *CPK6*, *ABI1, ABI2*, and *MYB44* in *poco1,* which are involved in stomatal regulation, may result in the stomatal closure not operating properly. This condition would consequently enhance the water loss in *poco1*, which does not allow drought tolerance. Furthermore, these results hint to the importance of ABA and ROS in control of stomatal function [159].

The expression of many transcription factors from different classes is altered in different developmental stages in *poco1*, suggesting that developmental processes in *poco1* are controlled by a complex transcriptional regulation. The ERF transcription factor family, which is implicated in the transcriptional regulation of diverse cellular functions related to growth and development, responds to environmental stimuli [160]. The bHLH transcription factors bHLH81 (*FBH2*) and bHLH136 (*PRE1*), NAC transcription factor (NAC089), RAV (TEM1), and ERF (RAV1) transcription factors were demonstrated to affect flowering. The WRKY and MYB transcription factors are reported to be involved in ABA signaling [88, 100]. The differentially regulated genes encoding transcription factors that are involved in ABA signaling are as follows: *WRKY2*, *WRKY33*, *WRKY25*, *WRKY46*, *MYB20*, *MYB32*, *MYB51*, *MYB73*, and *MYB44*. Other groups of differentially regulated transcription factors in *poco1* such as bZIP, CCCH zinc finger, C2H2 zinc finger, ERF, GATA, GRAS, Homeobox, and MYB-like are involved in several plant processes. The common biological processes associated with these regulatory proteins are involvement in stress and development regulation. The most probable explanation is the deficient ABA signaling in *poco1*, which affects stress tolerance and plant development. Besides, studies reported the significant involvement of NAC, CCCH zinc finger, bHLH, and WRKY transcription factors in modulating the stress response and flowering [85, 94, 161–163]. Based on the previous report [23], several transcription factors bind to transcripts from all five respiratory complexes in mitochondria and function as regulators of mitochondrial gene expression. Several genes encoding these transcription factors were found to be differentially expressed in *poco1* in our RNA-seq results, including *WRKY15*, *WRKY30, WRKY33*, *ABF4*, *Athb-6*, *bZIP10*, *bZIP25*, and *bHLH81*.

**Fig. 5:**
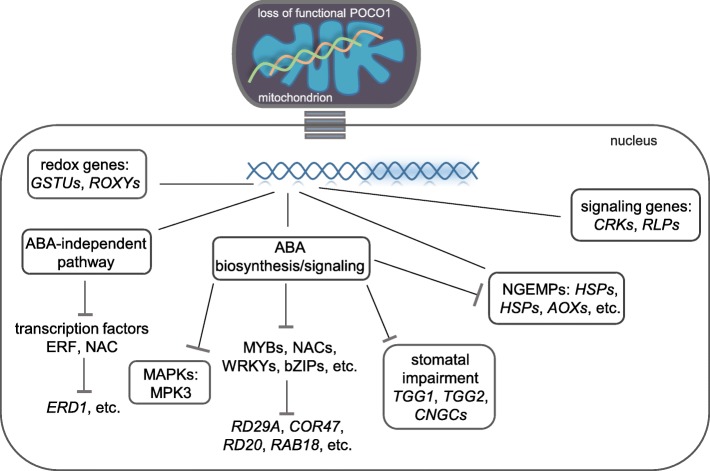
The possible regulatory network of the gene expression in response to the loss of function of POCO1 in mitochondria. Loss of function of POCO1 is sensed by mitochondria. Subsequently, a retrograde signaling cascade may be activated to launch the gene expression changes. Several transcription factors, which control stress-inducible gene expression are affected. Several processes may be under the influence of defected ABA signaling in poco1. Black lines indicate crosstalk and differential regulation. Dashed arrows are indicative of possible inhibiting effects

MAPK cascades are involved in the ABA signaling and stress tolerance and are triggered by a wide range of signals including ABA, auxin, ethylene, ROS, and pathogens [164]. Thus, the down-regulation of MAPK cascades may be affected by the ABA deficiency in *poco1* (Fig. 5). MAPKs frequently regulate a wide range of downstream events and thus define downstream signals. Therefore, any change in their expression may lead to changes in other signaling factors [165, 166]. RLPs are membrane-bound signaling molecules, which contain an extracellular receptor domain and can be transferred into the nucleus, chloroplast, or mitochondria. RLPs act to improve plant responses to biotic and abiotic stresses [167]. Alteration in the induction of genes such as *RLKs*, *CRKs*, MAPK cascades, *LRR*-*RKs*, and *RLPs* may emphasize the impact of *poco1* on cellular signaling.

Redox signals are involved in various aspects of plant biology and are specifically critical in cross-tolerance phenomena, allowing a general acclimation of plants to stressful conditions [24]. As genes related to the redox cascade such as *GRXs*, *GSTs, TRXs*, and *ROCs* were found to be differentially regulated, the redox state in *poco1* may be altered. This hypothesis is supported by the higher accumulation of ROS in *poco1* plants and the further fact that change in the ROS level leads to the redox state alteration [24]. Redox-based signaling is an attractive candidate to be a key constituent in the mitochondria-nucleus communication cascade [105, 114, 168, 169]. However, it has been proven that retrograde signaling exploits factors expanded in other contexts such as signaling factors associated with MAP kinase or ABA signaling [103]. Therefore, a general mitochondrial stress response appears linked to ABA signaling [170]. Glutathione s-transferases (GSTs) are a family of enzymes that catalyzes the conjugation of reduced GSH to a wide range of substrates and modulates GSH homeostasis to regulate development in *Arabidopsis* [171]. Previous studies demonstrated that *GST* transcripts are induced in plant tissues in response to the exogenous application of GSH, ABA, and ethylene [172, 173]. Thus, the lower induction of several *GSTs* in *poco1* may be supposedly due to ABA signaling and response defect. Stress-tolerant phenotypes, particularly drought stress, can be sufficiently explained by the greater glutathione (GSHs), ABA accumulation, and fine-tuned ABA signaling [174, 175]. Moreover, a lower GSH redox state was shown to lead to the early flowering in *Oncidium* orchid [176]. These pieces of evidence are consistent with early flowering and sensitivity to the drought stress phenotype of *poco1*.

In case of any change in the cellular or metabolic status in organelles (e.g., ROS accumulation), the transcript level is adjusted to optimize organellar proteome. Due to the involvement of NGEMPs to a wide range of mitochondrial perturbations, they represent different expression characteristics, and this condition points toward the possibility of the existence of multiple mitochondrial retrograde regulations [23]. As ABA affects the induction of *ALTERNATIVE OXIDASE 1a* (*AOX1a*) [177], and due to the presence of the potential binding sites of ABA-responsive elements in the promoter region of *AOX1a* [23, 177], repression of *AOX1a* might be under the influence of corrupted ABA signaling in *poco1* (Fig. 5). However, *aox1a* plants were shown to accumulate higher superoxide radicals and were more sensitive to drought and light stresses, which is consistent with the *poco1* phenotype [178, 179]. Enhanced induction of stress-responsive *AOX1d* suggests the activation of the compensatory AOX pathway in *poco1* mutant mitochondria. The alteration of expression of many genes in *poco1*, in particular, NGEMPs on one hand and the redox state genes, on the other hand, offers the possibility for the presence of retrograde signals from mitochondria to the nucleus.

## Conclusion

We used RNA-seq analysis to provide an overview of the global transcriptome changes in *poco1* during different developmental stages. Most of the differentially regulated genes were identified in inflorescence-inflorescence, and the result asserts the necessity of biological analysis in different developmental stages. Different biological processes were enriched in different comparisons, which indicates that various processes are involved in the regulation of *poco1*. Differentially expressed genes in diverse developmental stages associated with flowering, ABA signaling and response, drought and oxidative stresses, redox state, and genes associated with mitochondrial perturbation were identified. Based on the RNA-seq results, *poco1* leads to the differential regulation of some flowering genes, which affect flowering time promotion. Moreover, *poco1* considerably affects ABA signaling from its early signal transduction. Our data support the alteration of redox state in *poco1*. Furthermore, alteration in the redox state and NGEMPs expression highlights the presence of retrograde signals to transmit their functional status to regulate plant development. According to these data and our previous study, the impairment of mitochondrial function in *poco1* and a higher generation of ROS may cause redox imbalance, which affects the expression of many genes including ABA-, flowering-, and stress-associated genes and further causes lower tolerance to drought stress. Overall, the data generated in this study can be used to facilitate further investigations of the molecular mechanisms underlying flowering time and ABA signaling associated with mitochondrial proteins and improve the knowledge about the PPR protein family.

## Methods

### Plant materials

In this study, *Arabidopsis thaliana* plants wild-type, WS-4 (Wassilewskija), and *poco1* mutants (FLAG_465F03) [180] were used. Wild-type and FLAG_465F03 were obtained from the Versailles Arabidopsis Stock Center (INRA; http://publiclines.versailles.inra.fr/). Plants were grown under controlled conditions in the growth chamber at 22 °C with 65% relative humidity, a light intensity of 110 μmol m^− 2^ s^− 1^, and the photoperiod of 16 h light and 8 h dark. Samples for RNA isolation and RNA-seq analysis were harvested two and a half hours after the start of the day period (11:00 AM).

### RNA isolation and RNA-seq

Total RNA was isolated from wild-type and *poco1* plant leaves using TRIzol (Peqlab, Erlangen, Germany). RNA degradation and contamination were examined on a 2% agarose gel. Three biological replicates were performed for each stage for each genotype, for a total of 12 samples. RNA sequencing was performed by GATC Biotech AG (Konstanz, Germany) using Illumina technology by the HiSeq 2000 machine (125 bp paired-end reads). All steps performed have been developed and validated by GATC Biotech AG.

### Differential gene expression analysis

Obtained raw sequences were evaluated by the CLC Genomics Workbench 7.5.1 program from CLC Bio (Qiagen, Hilden) based on the principles of [181]. The RNA-seq data initially went through quality control and were trimmed. The trimmed sequences were mapped to the unmasked version of *Arabidopsis thaliana* (WS-0) reference genome from the 1001 Genomes Project (https://1001genomes.org/) (http://mtweb.cs.ucl.ac.uk/mus/www/19genomes/fasta/). The annotated gene model was used according to Arabidopsis genome annotation (TAIR10). Expression levels derived from the RNA-seq data Reads per Kilobase per Million mapped reads (RPKMs) [181] and fold changes were reported using CLC Genomics Workbench 7.5.1. CLC Genomic workbench 7.5.1 follows RNAseq protocol proposed by [181]. The false discovery rate (FDR) < 0.05 was chosen as the cut-off threshold for the identification of significant expression differences [182]. Differentially expressed genes were defined as those with a fold change either ≥2 or ≤ − 2.

### Gene ontology, Venn diagrams, and heat maps

The unique gene identifiers were obtained for each category and were then used for gene ontology (GO) enrichment analysis. The GO enrichment was performed with the set of background genes (those detected in each comparison) using the g:profiler online tool [183], and the Venn diagrams to show the overlapping genes of different comparisons were made by an online tool (http://bioinformatics.psb.ugent.be/webtools/Venn/). A cut-off value of the adjusted *p*-value (*P*adj) was used for the GO analysis. Fold changes (log10) were used for representing in heat maps.

### Coverage analysis of *POCO1*

The cDNA sequence of *POCO1* was acquired from NCBI (https://www.ncbi.nlm.nih.gov/) (GenBank Accession: NM_101417.4) and imported to the CLC Genomics Workbench 7.5.1 program. The “Map reads to contigs” tool from the CLC Genomics Workbench 7.5.1 program was used to map reads in wild-type and *poco1* mutants (*n* = 3 for each genotype) to the reference gene (*AT1G15480*), and the read depth was examined. Visual inspection was obtained by the CLC Genomics Workbench 7.5.1 program.

The datasets supporting the conclusions of this article are included within the article and its additional files.

## Supplementary information

**Additional file 1: Table S1, Table S2** and **Table S3.** List of differentially expressed genes. Differentially expressed genes in *poco1* in pre-inflorescence-inflorescence, inflorescence-flowering and inflorescence-inflorescence (fold changes either ≥2 or ≤ − 2 FDR cutoff < 0.05).

**Additional file 2: Figure S1.** GO enrichment terms. Top 5 molecular functions (GO:MF) and top 30 biological processes (GO:BP) for up- and down-regulated genes in pre-inflorescence-inflorescence, inflorescence-flowering and inflorescence-inflorescence are shown. The adjusted *p*-values (*P*adj) are shown in negative log10 scale.

**Additional file 3: Figure S2.** Expression of flowering-related genes affected by *poco1*. Heat map of flowering-related genes. Differentially expressed flowering genes in *poco1* versus wild-type in three comparisons are shown. Altered expression of flowering-related genes may explain the early-flowering phenotype of poco1. Fold changes (log10) were used for representing in the heat map. Red and blue represent up- and down-regulated transcripts respectively. Black represents that fold changes either ≥2 or ≤ − 2 with an FDR < 0.05 were not detected. Fold changes are relative to wild-type.

**Additional file 4: Figure S3.***poco1* impaired ABA signaling and response. Heat map of ABA-related differentially expressed genes. poco1 repressed numerous ABA-related genes, which results in ABA signaling deficiency. Fold changes (log10) were used for representing in the heat map. Red and blue represent up- and down-regulated transcripts respectively. Black represents that fold changes either ≥2 or ≤ − 2 with an FDR < 0.05 were not detected. Fold changes are relative to wild-type.

**Additional file 5: Figure S4.** Fold change expression of drought and oxidative stress genes. Fold change heat map of the differentially expressed drought and oxidative stress-related genes in *poco1*. Fold changes (log10) were used for representing in the heat map. Red and blue represent up- and down-regulated transcripts respectively. Black represents that fold changes either ≥2 or ≤ − 2 with an FDR < 0.05 were not detected.

**Additional file 6: Figure S5.** Fold change heat map of genes encoding transcription factors. Fold change expression of genes encoding transcription factors is visualized by a heat map. The expression of different classes of transcription factors is affected in *poco1*. Fold changes (log10) were used for representing in the heat map. Red and blue represent up- and down-regulated transcripts respectively. Black represents that fold changes either ≥2 or ≤ − 2 with an FDR < 0.05 were not detected. TFs, transcription factors.

**Additional file 7: Figure S6.** Alteration of expression of genes associated with cellular signaling and mitochondrial perturbation targets in *poco1*. Genes associated with cellular signaling and mitochondrial perturbation were found differentially regulated in *poco1*. Fold changes (log10) were used for representing in the heat map. Red and blue represent up- and down-regulated transcripts respectively. Black represents that fold changes either ≥2 or ≤ − 2 with an FDR < 0.05 were not detected. Fold changes are relative to wild-type.

**Additional file 8: Figure S7.** Expression alteration in redox-related genes and genes associated with stomatal function. Genes associated with the redox status and stomatal function were found to have differential regulation in *poco1*. Fold changes (log10) were used for representing in heat maps. Red and blue represent up- and down-regulated transcripts respectively. Black represents that fold changes either ≥2 or ≤ − 2 with an FDR < 0.05 were not detected. Fold changes are relative to wild-type.

## Data Availability

The datasets generated and analyzed during the current study are available in the [ArrayExpress database at EMBL-EBI (www.ebi.ac.uk/arrayexpress)] repository under accession number E-MTAB-8912 (http://www.ebi.ac.uk/arrayexpress/experiments/E-MTAB-8912/).

## Abbreviations

ABA: Abscisic acid
ABFs: ABRE-binding factors
AREBs: ABRE-binding proteins
ABRE: ABA-responsive element
*A. thaliana*: *Arabidopsis thaliana*
FDR: False discovery rate
FT: FLOWERING LOCUS T
GO: Gene ontology
GRXs: Glutaredoxins
GSTs: Glutathione s-transferases
MAPKs: Mitogen-activated protein kinases
NGEMPs: Nuclear genes encoded mitochondrial proteins
POCO1: PRECOCIOUS1
PPR: Pentatricopeptide repeat
PYR1/PYL/RCAR: Pyrabactin resistance 1/ PYR1-like/regulatory components of ABA receptors
ROS: Reactive oxygen species
RPKMs: Reads per kilobase per million mapped reads
SnRK2: Sucrose nonfermenting 1-related protein kinases 2
TF: Transcription factors
TRXs: Thioredoxin
