# Supplementary material for: Transcriptomic analysis of poco1, a mitochondrial pentatricopeptide repeat protein mutant in Arabidopsis thaliana
Source: BMC Plant Biol. 2020 May 12;20:209. doi: 10.1186/s12870-020-02418-z (PMC7216612; doi:10.1186/s12870-020-02418-z)
Supplement: Supplementary file 2 — Additional file 2: Figure S1. GO enrichment terms. Top 5 molecular functions (GO:MF) and top 30 biological processes (GO:BP) for up- and down-regulated genes in pre-inflorescence-inflorescence, inflorescence-flowering and inflorescence-inflorescence are shown. The adjusted p-values (Padj) are shown in negative log10 scale. [file 12870_2020_2418_MOESM2_ESM.doc]

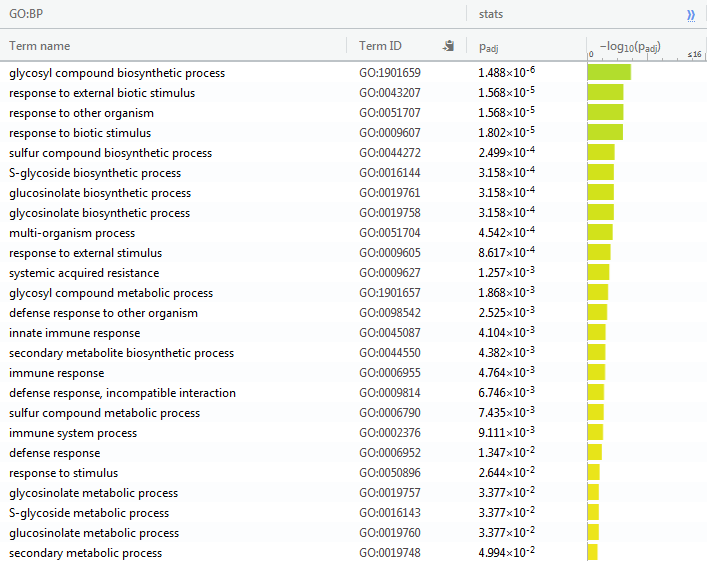


**GO analysis of up-regulated genes in comparison 1 (pre-inflorescence-inflorescence)**


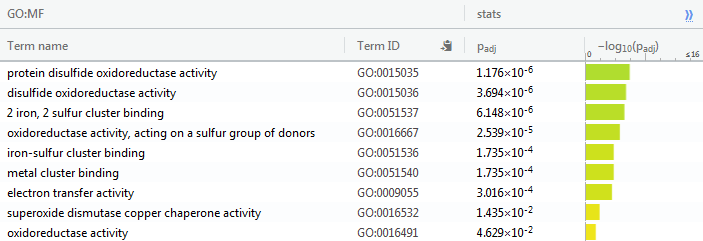

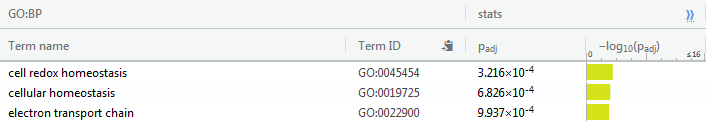


**GO analysis of down-regulated genes in comparison 1 (pre-inflorescence-inflorescence)**


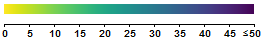


The colors for log scale


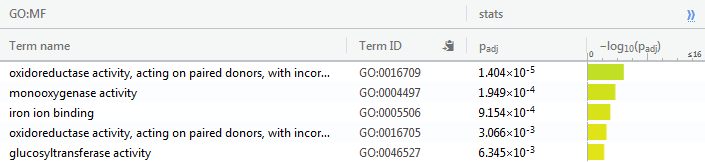

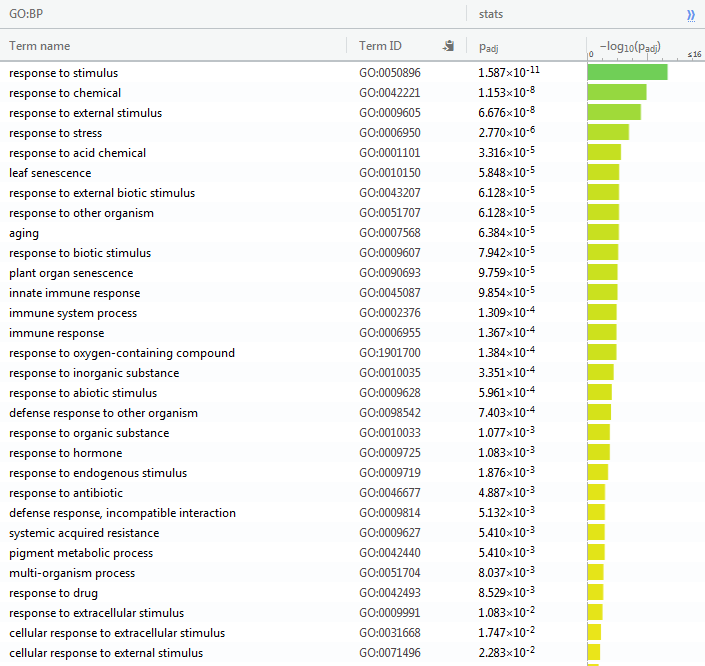


**GO analysis of up-regulated genes in comparison 2 (inflorescence-flowering)**


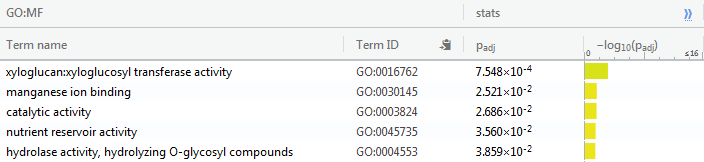

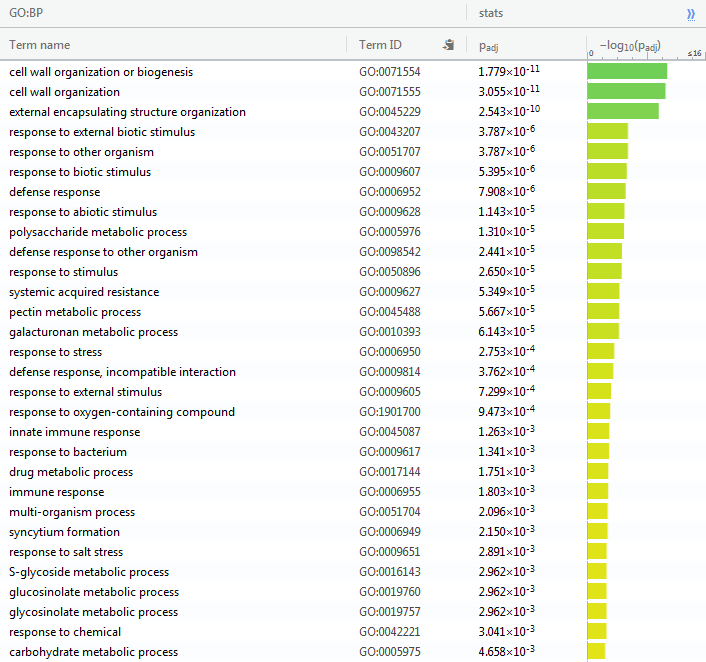


**GO analysis of down-regulated genes in comparison 2 (inflorescence-flowering)**


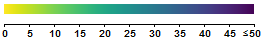


The colors for log scale


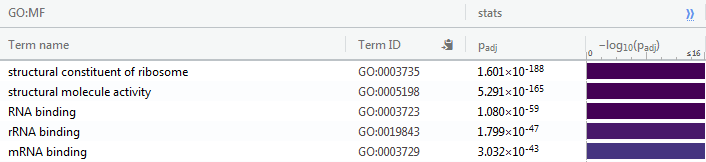

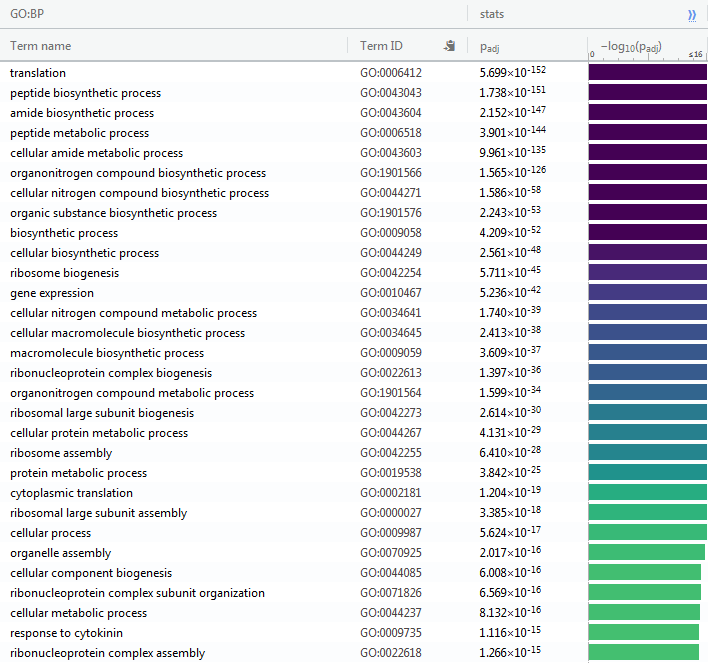


**GO analysis of up-regulated genes in comparison 3 (inflorescence-inflorescence)**


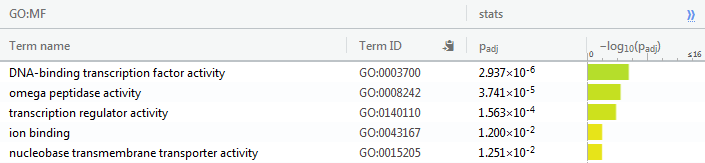

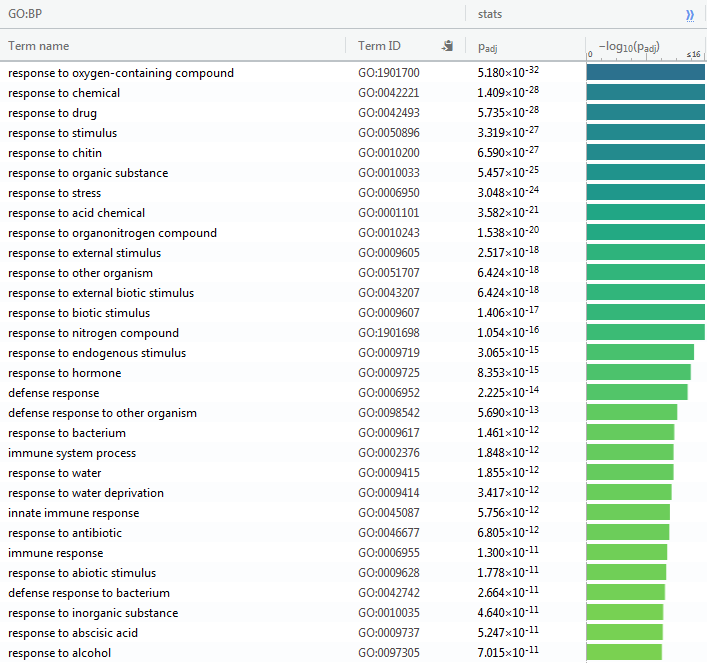


**GO analysis of down-regulated genes in comparison 3 (inflorescence-inflorescence)**


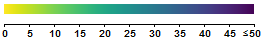


The colors for log scale

**Fig. S1**
