# Supplementary material for: Transcriptomic analysis of poco1, a mitochondrial pentatricopeptide repeat protein mutant in Arabidopsis thaliana
Source: BMC Plant Biol. 2020 May 12;20:209. doi: 10.1186/s12870-020-02418-z (PMC7216612; doi:10.1186/s12870-020-02418-z)
Supplement: Supplementary file 3 — Additional file 3: Figure S2. Expression of flowering-related genes affected by poco1. Heat map of flowering-related genes. Differentially expressed flowering genes in poco1 versus wild-type in three comparisons are shown. Altered expression of flowering-related genes may explain the early-flowering phenotype of poco1. Fold changes (log10) were used for representing in the heat map. Red and blue represent up- and down-regulated transcripts respectively. Black represents that fold changes either ≥2 or ≤ − 2 with an FDR < 0.05 were not detected. Fold changes are relative to wild-type. [file 12870_2020_2418_MOESM3_ESM.ppt]

## Slide 1
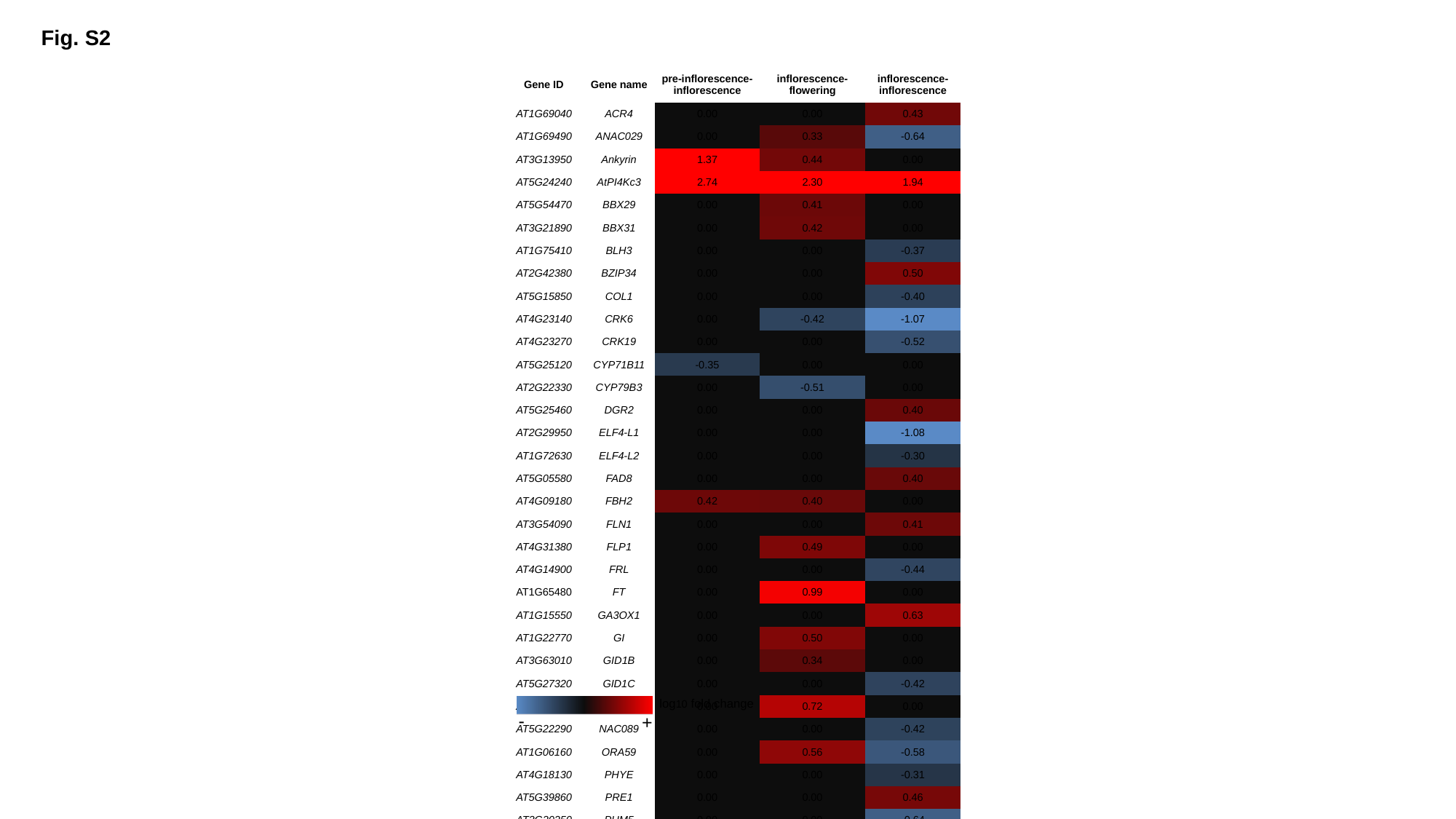

Fig. S2
| Gene ID | Gene name | pre-inflorescence-inflorescence | inflorescence-flowering | inflorescence-inflorescence |
| --- | --- | --- | --- | --- |
| AT1G69040 | ACR4 | 0.00 | 0.00 | 0.43 |
| AT1G69490 | ANAC029 | 0.00 | 0.33 | -0.64 |
| AT3G13950 | Ankyrin | 1.37 | 0.44 | 0.00 |
| AT5G24240 | AtPI4Kc3 | 2.74 | 2.30 | 1.94 |
| AT5G54470 | BBX29 | 0.00 | 0.41 | 0.00 |
| AT3G21890 | BBX31 | 0.00 | 0.42 | 0.00 |
| AT1G75410 | BLH3 | 0.00 | 0.00 | -0.37 |
| AT2G42380 | BZIP34 | 0.00 | 0.00 | 0.50 |
| AT5G15850 | COL1 | 0.00 | 0.00 | -0.40 |
| AT4G23140 | CRK6 | 0.00 | -0.42 | -1.07 |
| AT4G23270 | CRK19 | 0.00 | 0.00 | -0.52 |
| AT5G25120 | CYP71B11 | -0.35 | 0.00 | 0.00 |
| AT2G22330 | CYP79B3 | 0.00 | -0.51 | 0.00 |
| AT5G25460 | DGR2 | 0.00 | 0.00 | 0.40 |
| AT2G29950 | ELF4-L1 | 0.00 | 0.00 | -1.08 |
| AT1G72630 | ELF4-L2 | 0.00 | 0.00 | -0.30 |
| AT5G05580 | FAD8 | 0.00 | 0.00 | 0.40 |
| AT4G09180 | FBH2 | 0.42 | 0.40 | 0.00 |
| AT3G54090 | FLN1 | 0.00 | 0.00 | 0.41 |
| AT4G31380 | FLP1 | 0.00 | 0.49 | 0.00 |
| AT4G14900 | FRL | 0.00 | 0.00 | -0.44 |
| AT1G65480 | FT | 0.00 | 0.99 | 0.00 |
| AT1G15550 | GA3OX1 | 0.00 | 0.00 | 0.63 |
| AT1G22770 | GI | 0.00 | 0.50 | 0.00 |
| AT3G63010 | GID1B | 0.00 | 0.34 | 0.00 |
| AT5G27320 | GID1C | 0.00 | 0.00 | -0.42 |
| AT2G21660 | GRP7 | 0.00 | 0.72 | 0.00 |
| AT5G22290 | NAC089 | 0.00 | 0.00 | -0.42 |
| AT1G06160 | ORA59 | 0.00 | 0.56 | -0.58 |
| AT4G18130 | PHYE | 0.00 | 0.00 | -0.31 |
| AT5G39860 | PRE1 | 0.00 | 0.00 | 0.46 |
| AT3G20250 | PUM5 | 0.00 | 0.00 | -0.64 |
| AT1G13260 | RAV1 | 0.00 | 0.00 | -0.41 |
| AT5G14070 | ROXY2 | 0.00 | 0.00 | 1.54 |
| AT1G25560 | TEM1 | 0.00 | 0.00 | -0.37 |
| AT2G43510 | TI1 | 0.00 | 0.60 | 0.00 |
| AT1G78190 | TRM112A | 0.00 | 0.40 | 0.00 |
| AT2G30140 | UGT87A2 | 0.70 | 0.47 | 0.37 |
| AT1G09390 | AT1G09390 | 0.00 | 0.00 | 0.47 |
| AT1G80940 | AT1G80940 | 0.00 | 0.00 | -0.37 |
| AT5G56120 | AT5G56120 | 0.00 | 0.00 | 0.43 |
log10 fold change
-
+
