# Supplementary material for: Transcriptomic analysis of poco1, a mitochondrial pentatricopeptide repeat protein mutant in Arabidopsis thaliana
Source: BMC Plant Biol. 2020 May 12;20:209. doi: 10.1186/s12870-020-02418-z (PMC7216612; doi:10.1186/s12870-020-02418-z)
Supplement: Supplementary file 4 — Additional file 4: Figure S3.poco1 impaired ABA signaling and response. Heat map of ABA-related differentially expressed genes. poco1 repressed numerous ABA-related genes, which results in ABA signaling deficiency. Fold changes (log10) were used for representing in the heat map. Red and blue represent up- and down-regulated transcripts respectively. Black represents that fold changes either ≥2 or ≤ − 2 with an FDR < 0.05 were not detected. Fold changes are relative to wild-type. [file 12870_2020_2418_MOESM4_ESM.ppt]

## Slide 1
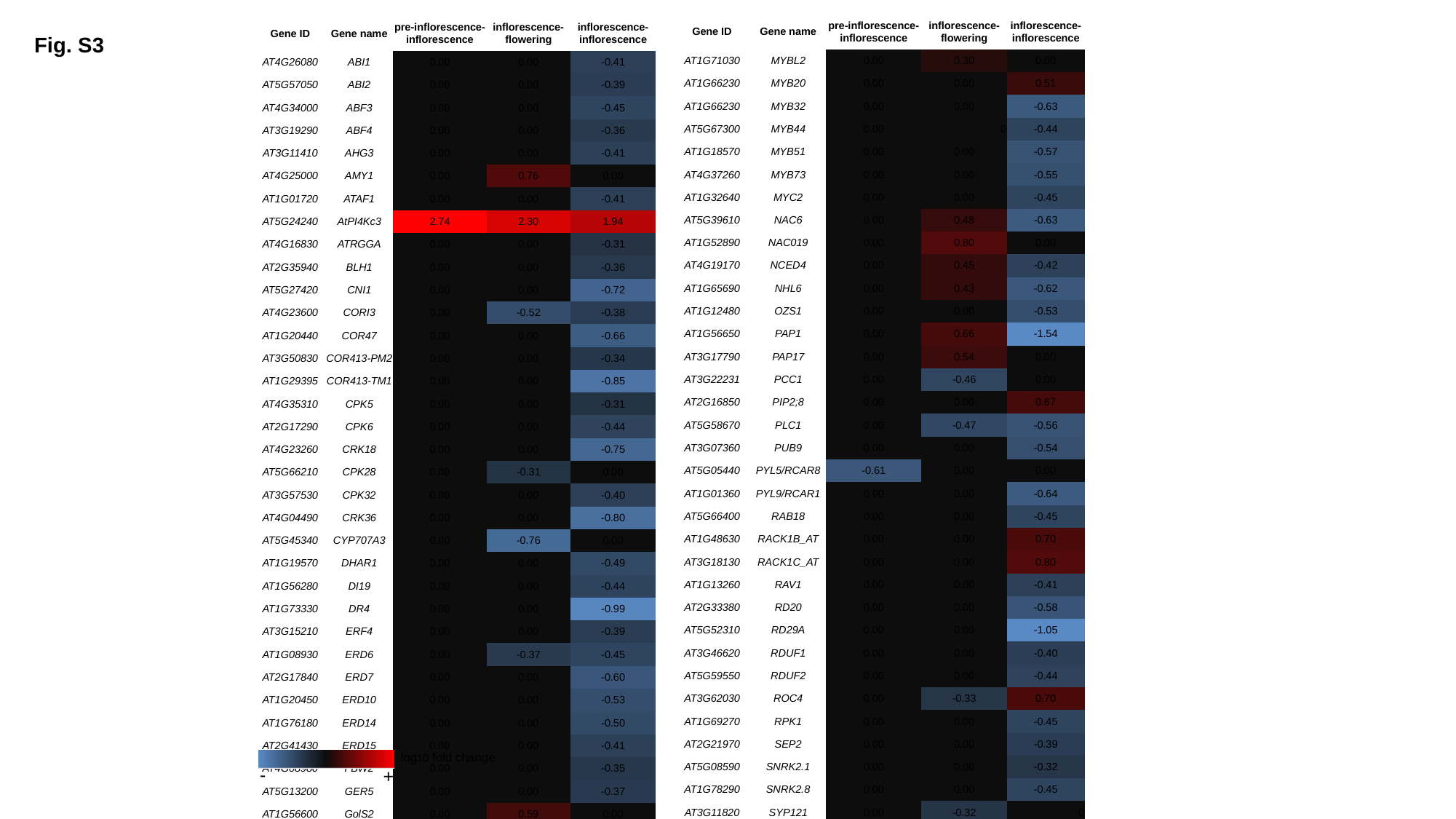

| Gene ID | Gene name | pre-inflorescence-inflorescence | inflorescence-flowering | inflorescence-inflorescence |
| --- | --- | --- | --- | --- |
| AT1G71030 | MYBL2 | 0.00 | 0.30 | 0.00 |
| AT1G66230 | MYB20 | 0.00 | 0.00 | 0.51 |
| AT1G66230 | MYB32 | 0.00 | 0.00 | -0.63 |
| AT5G67300 | MYB44 | 0.00 | 0 | -0.44 |
| AT1G18570 | MYB51 | 0.00 | 0.00 | -0.57 |
| AT4G37260 | MYB73 | 0.00 | 0.00 | -0.55 |
| AT1G32640 | MYC2 | 0.00 | 0.00 | -0.45 |
| AT5G39610 | NAC6 | 0.00 | 0.48 | -0.63 |
| AT1G52890 | NAC019 | 0.00 | 0.80 | 0.00 |
| AT4G19170 | NCED4 | 0.00 | 0.45 | -0.42 |
| AT1G65690 | NHL6 | 0.00 | 0.43 | -0.62 |
| AT1G12480 | OZS1 | 0.00 | 0.00 | -0.53 |
| AT1G56650 | PAP1 | 0.00 | 0.66 | -1.54 |
| AT3G17790 | PAP17 | 0.00 | 0.54 | 0.00 |
| AT3G22231 | PCC1 | 0.00 | -0.46 | 0.00 |
| AT2G16850 | PIP2;8 | 0.00 | 0.00 | 0.67 |
| AT5G58670 | PLC1 | 0.00 | -0.47 | -0.56 |
| AT3G07360 | PUB9 | 0.00 | 0.00 | -0.54 |
| AT5G05440 | PYL5/RCAR8 | -0.61 | 0.00 | 0.00 |
| AT1G01360 | PYL9/RCAR1 | 0.00 | 0.00 | -0.64 |
| AT5G66400 | RAB18 | 0.00 | 0.00 | -0.45 |
| AT1G48630 | RACK1B\_AT | 0.00 | 0.00 | 0.70 |
| AT3G18130 | RACK1C\_AT | 0.00 | 0.00 | 0.80 |
| AT1G13260 | RAV1 | 0.00 | 0.00 | -0.41 |
| AT2G33380 | RD20 | 0.00 | 0.00 | -0.58 |
| AT5G52310 | RD29A | 0.00 | 0.00 | -1.05 |
| AT3G46620 | RDUF1 | 0.00 | 0.00 | -0.40 |
| AT5G59550 | RDUF2 | 0.00 | 0.00 | -0.44 |
| AT3G62030 | ROC4 | 0.00 | -0.33 | 0.70 |
| AT1G69270 | RPK1 | 0.00 | 0.00 | -0.45 |
| AT2G21970 | SEP2 | 0.00 | 0.00 | -0.39 |
| AT5G08590 | SNRK2.1 | 0.00 | 0.00 | -0.32 |
| AT1G78290 | SNRK2.8 | 0.00 | 0.00 | -0.45 |
| AT3G11820 | SYP121 | 0.00 | -0.32 | 0 |
| AT5G37770 | TCH2 | 0.00 | -0.44 | 0 |
| AT2G41100 | TCH3 | 0.00 | -0.61 | -0.40 |
| AT5G26000 | TGG1 | 0.00 | -1.28 | 0.53 |
| AT5G25980 | TGG2 | -2.16 | -4.24 | -1.86 |
| AT3G50740 | UGT72E1 | 0.00 | 0.32 | -0.40 |
| AT4G32150 | VAMP711 | 0.00 | 0.00 | -0.44 |
| AT5G56270 | WRKY2 | 0.00 | 0.00 | -0.45 |
| AT2G30250 | WRKY25 | 0.00 | 0.00 | -0.55 |
| AT2G38470 | WRKY33 | 0.00 | 0.00 | -0.60 |
| AT2G46400 | WRKY46 | 0.00 | 0.00 | -0.64 |
| AT2G28200 | ZAT10 | 0.00 | 0.00 | -0.33 |
| AT5G67450 | ZF1 | 0.00 | -0.50 | 0.00 |
| AT3G19580 | ZF2 | 0.00 | 0.00 | -0.61 |
| AT1G13930 | AT1G13930 | 0.00 | 0.00 | -0.37 |
| AT1G63840 | AT1G63840 | 0.00 | 0.00 | -0.82 |
| AT1G70810 | AT1G70810 | 0.00 | 0.00 | -0.56 |
| AT2G47710 | AT2G47710 | 0.00 | 0.00 | -0.34 |
| AT3G09020 | AT3G09020 | 0.00 | 0.00 | -0.68 |
| AT5G04760 | AT5G04760 | 0.00 | 0.00 | -0.60 |
| Gene ID | Gene name | pre-inflorescence-inflorescence | inflorescence-flowering | inflorescence-inflorescence |
| --- | --- | --- | --- | --- |
| AT4G26080 | ABI1 | 0.00 | 0.00 | -0.41 |
| AT5G57050 | ABI2 | 0.00 | 0.00 | -0.39 |
| AT4G34000 | ABF3 | 0.00 | 0.00 | -0.45 |
| AT3G19290 | ABF4 | 0.00 | 0.00 | -0.36 |
| AT3G11410 | AHG3 | 0.00 | 0.00 | -0.41 |
| AT4G25000 | AMY1 | 0.00 | 0.76 | 0.00 |
| AT1G01720 | ATAF1 | 0.00 | 0.00 | -0.41 |
| AT5G24240 | AtPI4Kc3 | 2.74 | 2.30 | 1.94 |
| AT4G16830 | ATRGGA | 0.00 | 0.00 | -0.31 |
| AT2G35940 | BLH1 | 0.00 | 0.00 | -0.36 |
| AT5G27420 | CNI1 | 0.00 | 0.00 | -0.72 |
| AT4G23600 | CORI3 | 0.00 | -0.52 | -0.38 |
| AT1G20440 | COR47 | 0.00 | 0.00 | -0.66 |
| AT3G50830 | COR413-PM2 | 0.00 | 0.00 | -0.34 |
| AT1G29395 | COR413-TM1 | 0.00 | 0.00 | -0.85 |
| AT4G35310 | CPK5 | 0.00 | 0.00 | -0.31 |
| AT2G17290 | CPK6 | 0.00 | 0.00 | -0.44 |
| AT4G23260 | CRK18 | 0.00 | 0.00 | -0.75 |
| AT5G66210 | CPK28 | 0.00 | -0.31 | 0.00 |
| AT3G57530 | CPK32 | 0.00 | 0.00 | -0.40 |
| AT4G04490 | CRK36 | 0.00 | 0.00 | -0.80 |
| AT5G45340 | CYP707A3 | 0.00 | -0.76 | 0.00 |
| AT1G19570 | DHAR1 | 0.00 | 0.00 | -0.49 |
| AT1G56280 | DI19 | 0.00 | 0.00 | -0.44 |
| AT1G73330 | DR4 | 0.00 | 0.00 | -0.99 |
| AT3G15210 | ERF4 | 0.00 | 0.00 | -0.39 |
| AT1G08930 | ERD6 | 0.00 | -0.37 | -0.45 |
| AT2G17840 | ERD7 | 0.00 | 0.00 | -0.60 |
| AT1G20450 | ERD10 | 0.00 | 0.00 | -0.53 |
| AT1G76180 | ERD14 | 0.00 | 0.00 | -0.50 |
| AT2G41430 | ERD15 | 0.00 | 0.00 | -0.41 |
| AT4G08980 | FBW2 | 0.00 | 0.00 | -0.35 |
| AT5G13200 | GER5 | 0.00 | 0.00 | -0.37 |
| AT1G56600 | GolS2 | 0.00 | 0.59 | 0.00 |
| AT3G20470 | GRP5 | 0.00 | -0.50 | 0.36 |
| AT2G32690 | GRP23 | 0.00 | -0.39 | 0.00 |
| AT1G07430 | HAI2 | 0.00 | 0.65 | 0.00 |
| AT1G72770 | HAB1 | 0.00 | 0.00 | -0.42 |
| AT1G17550 | HAB2 | 0.00 | 0.00 | -0.42 |
| AT2G22430 | HB6 | 0.00 | 0.00 | -0.40 |
| AT3G61890 | HB-12 | 0.00 | 0.32 | -0.56 |
| AT1G74520 | HVA22A | 0.00 | 0.00 | -0.31 |
| AT5G06760 | LEA4-5 | 0.00 | 0.00 | -0.81 |
| AT1G01470 | LEA14 | 0.00 | 0.00 | -0.31 |
| AT5G59320 | LTP3 | -0.72 | -0.47 | -0.98 |
| AT5G59310 | LTP4 | -0.97 | -0.43 | -1.35 |
| AT5G52900 | MAKR6 | 0.00 | 0.36 | 0.00 |
| AT3G63210 | MARD1 | 0.00 | 0.00 | -0.54 |
| AT1G70890 | MLP43 | 0.00 | -0.32 | -0.48 |
| AT1G70000 | MYBD | 0.00 | 0.31 | 0.00 |
| AT5G67300 | MYBR1 | 0.00 | 0.00 | -0.44 |
| AT2G47190 | MYB2 | 0.00 | 1.01 | 0.00 |
Fig. S3
log10 fold change
-
+
