# Supplementary material for: Transcriptomic analysis of poco1, a mitochondrial pentatricopeptide repeat protein mutant in Arabidopsis thaliana
Source: BMC Plant Biol. 2020 May 12;20:209. doi: 10.1186/s12870-020-02418-z (PMC7216612; doi:10.1186/s12870-020-02418-z)
Supplement: Supplementary file 5 — Additional file 5: Figure S4. Fold change expression of drought and oxidative stress genes. Fold change heat map of the differentially expressed drought and oxidative stress-related genes in poco1. Fold changes (log10) were used for representing in the heat map. Red and blue represent up- and down-regulated transcripts respectively. Black represents that fold changes either ≥2 or ≤ − 2 with an FDR < 0.05 were not detected. [file 12870_2020_2418_MOESM5_ESM.doc]

| **Gene ID** | **Gene name** | **pre-inflorescence-inflorescence** | | **inflorescence-flowering** | **inflorescence-inflorescence** |
| --- | --- | --- | --- | --- | --- |
| *AT4G34000* | *ABF3* | 0.00 | 0.00 | | -0.45 |
| *AT3G19290* | *ABF4* | 0.00 | 0.00 | | -0.36 |
| *AT5G06530* | *ABCG22* | 0.00 | 0.00 | | -0.46 |
| *AT4G34710* | *ADC2* | 0.00 | 0.00 | | 0.34 |
| *AT4G19710* | *AK-HSDH II* | 0.00 | 0.00 | | 0.36 |
| *AT3G29035* | *ANAC059* | 0.00 | 0.72 | | -0.96 |
| *AT5G65020* | *ANNAT2* | 0.00 | -0.54 | | 0.00 |
| *AT1G35720* | *ANNAT1* | 0.00 | -0.48 | | 0.00 |
| *AT2G32800* | *AP4.3A* | 0.00 | 0.00 | | -0.34 |
| *AT5G43850* | *ARD4* | 0.00 | 0.32 | | 0.00 |
| *AT5G03630* | *ATMDAR2* | 0.00 | 0.00 | | -0.47 |
| *AT5G28540* | *BIP1* | 0.30 | 0.00 | | 0.00 |
| *AT5G42020* | *BIP2* | 0.00 | 0.00 | | 0.30 |
| *AT5G20230* | *BCB* | 0.00 | 0.00 | | -0.43 |
| *AT3G50930* | *BCS1* | 0.00 | 0.00 | | -0.53 |
| *AT4G25700* | *BETA-OHASE 1* | 0.00 | 0.00 | | 0.35 |
| *AT2G35940* | *BLH1* | 0.00 | 0.00 | | -0.36 |
| *AT5G44070* | *CAD1* | 0.00 | 0.00 | | -0.36 |
| *AT4G34230* | *CAD5* | 0.00 | 0.37 | | 0.00 |
| *AT4G39330* | *CAD9* | 0.00 | -0.58 | | 0.00 |
| *AT1G20620* | *CAT3* | 0.00 | 0.40 | | 0.00 |
| *AT5G48810* | *CB5-D* | 0.00 | 0.00 | | -0.31 |
| *AT5G53560* | *CB5-E* | 0.00 | 0.00 | | -0.33 |
| *AT1G12520* | *CCS* | -0.56 | 0.00 | | 0.00 |
| *AT4G33790* | *CER4* | 0.00 | 0.00 | | 0.73 |
| *AT5G24090* | *CHIA* | 0.00 | 0.92 | | 0.00 |
| *AT1G20440* | *COR47* | 0.00 | 0.00 | | -0.66 |
| *AT3G50830* | *COR413-PM2* | 0.00 | 0.00 | | -0.34 |
| *AT4G24280* | *cpHsc70-1* | 0.00 | 0.00 | | 0.42 |
| *AT5G49910* | *cpHsc70-2* | 0.00 | 0.00 | | 0.47 |
| *AT5G20720* | *CPN20* | 0.00 | 0.00 | | 0.45 |
| *AT2G28000* | *CPN60A* | 0.00 | 0.00 | | 0.70 |
| *AT3G13470* | *Cpn60beta2* | 0.00 | 0.00 | | 0.72 |
| *AT1G55490* | *CPN60B* | 0.00 | 0.00 | | 0.90 |
| *AT4G23190* | *CRK11* | 0.00 | -0.48 | | 0.00 |
| *AT4G23220* | *CRK14* | 0.00 | -0.49 | | 0.00 |
| *AT2G04030* | *CR88* | 0.00 | 0.00 | | 0.34 |
| *AT2G28190* | *CSD2* | -0.79 | -0.71 | | -0.47 |
| *AT2G45510* | *CYP704A2* | 0.00 | 0.00 | | -0.53 |
| *AT2G27010* | *CYP705A9* | 0.00 | 2.50 | | 0.00 |
| *AT3G20100* | *CYP705A19* | 0.00 | 0.36 | | 0.00 |
| *AT3G20130* | *CYP705A22* | 0.00 | -0.44 | | 0.00 |
| *AT4G22710* | *CYP706A2* | 0.00 | -0.63 | | -1.30 |
| *AT4G12300* | *CYP706A4* | 0.00 | 0.48 | | 0.00 |
| *AT4G12310* | *CYP706A5* | 0.00 | 0.39 | | 0.00 |
| *AT3G26220* | *CYP71B3* | 0.00 | 0.32 | | -0.58 |
| *AT3G26280* | *CYP71B4* | 0.00 | 0.31 | | -0.46 |
| *AT1G13110* | *CYP71B7* | 0.00 | 0.00 | | -0.32 |
| *AT5G25120* | *CYP71B11* | 0.00 | 0.00 | | -0.54 |
| *AT5G25140* | *CYP71B13* | 0.00 | 0.00 | | -0.38 |
| *AT3G26170* | *CYP71B19* | 0.00 | 0.56 | | -0.70 |
| *AT3G26180* | *CYP71B20* | 0.00 | 0.32 | | 0.00 |
| *AT3G26210* | *CYP71B23* | 0.00 | 0.00 | | -0.44 |
| *AT3G26290* | *CYP71B26* | 0.00 | 0.00 | | -0.97 |
| *AT3G26230* | *CYP71B24* | 0.50 | 0.00 | | 0.00 |
| *AT3G14620* | *CYP72A8* | 0.00 | -0.32 | | 0.00 |
| *AT3G10570* | *CYP77A6* | 0.00 | -0.56 | | 0.00 |
| *AT2G46660* | *CYP78A6* | 0.00 | 0.45 | | 0.00 |
| *AT3G61880* | *CYP78A9* | 0.00 | 0.52 | | 0.00 |
| *AT2G22330* | *CYP79B3* | 0.00 | -0.51 | | 0.00 |
| *AT1G16410* | *CYP79F1* | 0.41 | 0.00 | | 0.00 |
| *AT5G36220* | *CYP81D1* | 0.00 | 0.00 | | -0.49 |
| *AT5G57220* | *CYP81F2* | 0.00 | -0.67 | | -0.53 |
| *AT5G10610* | *CYP81K1* | 0.00 | 0.00 | | -0.44 |
| *AT4G00360* | *CYP86A2* | 0.00 | -0.33 | | 0.00 |
| *AT2G45970* | *CYP86A8* | 0.00 | -0.36 | | 0.00 |
| *AT1G64900* | *CYP89A2* | 0.00 | 0.00 | | -0.32 |
| *AT1G64950* | *CYP89A5* | 0.00 | 0.43 | | 0.00 |
| *AT3G03470* | *CYP89A9* | 0.00 | 0.64 | | -0.81 |
| *AT5G52320* | *CYP96A4* | 0.00 | -0.35 | | -0.47 |
| *AT1G19570* | *DHAR1* | 0.00 | 0.00 | | -0.49 |
| *AT1G56280* | *DI19* | 0.00 | 0.00 | | -0.44 |
| *AT5G24530* | *DMR6* | 0.63 | 0.00 | | 0.00 |
| *AT1G73330* | *DR4* | 0.00 | 0.00 | | -0.99 |
| *AT5G17710* | *EMB1241* | 0.00 | 0.00 | | 0.51 |
| *AT5G51070* | *ERD1* | 0.00 | 0.00 | | -0.53 |
| *AT3G30775* | *ERD5* | 0.00 | 0.56 | | 0.00 |
| *AT1G10370* | *ERD9* | 0.00 | -0.39 | | 0.32 |
| *AT1G20450* | *ERD10* | 0.00 | 0.00 | | -0.53 |
| *AT1G76180* | *ERD14* | 0.00 | 0.00 | | -0.50 |
| *AT2G41430* | *ERD15* | 0.00 | 0.00 | | -0.41 |
| *AT4G17490* | *ERF6* | 0.00 | -0.93 | | -0.86 |
| *AT3G56090* | *FER3* | 0.00 | 0.00 | | 0.83 |
| *AT5G23980* | *FRO4* | 0.00 | 1.00 | | 0.00 |
| *AT5G49730* | *FRO6* | 0.31 | 0.47 | | 0.00 |
| *AT5G49740* | *FRO7* | 0.00 | 0.00 | | -0.52 |
| *AT3G26650* | *GAPA* | 0.00 | 0.00 | | 0.37 |
| *AT1G12900* | *GAPA-2* | 0.00 | -0.35 | | 0.39 |
| *AT1G42970* | *GAPB* | 0.00 | 0.00 | | 0.38 |
| *AT1G15550* | *GA3OX1* | 0.00 | 0.00 | | 0.63 |
| *AT4G33010* | *GLDP1* | 0.00 | 0.34 | | 0.00 |
| *AT1G11840* | *GLX1* | 0.00 | -0.31 | | 0.00 |
| *AT1G56600* | *GolS2* | 0.00 | 0.59 | | -1.58 |
| *AT2G41540* | *GPDHC1* | 0.00 | -0.53 | | 0.00 |
| *AT2G21660* | *GRP7* | 0.00 | 0.72 | | 0.00 |
| *AT2G30860* | *GSTF9* | 0.35 | 0.00 | | 0.00 |
| *AT3G03190* | *GSTF11* | 0.00 | -0.37 | | -0.32 |
| *AT5G17220* | *GSTF12* | 0.00 | 1.01 | | 0.00 |
| *AT2G29460* | *GSTU4* | 0.00 | 0.58 | | -1.22 |
| *AT2G29420* | *GSTU7* | 0.00 | 0.00 | | -0.39 |
| *AT1G27130* | *GSTU13* | 0.00 | 0.00 | | -0.35 |
| *AT1G59670* | *GSTU15* | 0.00 | 0.40 | | -1.35 |
| *AT1G59700* | *GSTU16* | 0.00 | 0.00 | | -0.45 |
| *AT1G78370* | *GSTU20* | 0.00 | -0.53 | | 0.00 |
| *AT1G17170* | *GSTU24* | 0.00 | 0.00 | | -1.20 |
| *AT2G46680* | *HB-7* | 0.00 | 0.36 | | 0.00 |
| *AT3G61890* | *HB-12* | 0.00 | 0.32 | | -0.56 |
| *AT1G58290* | *HEMA1* | 0.00 | 0.00 | | -0.32 |
| *AT4G15440* | *HPL1* | 0.95 | 0.36 | | 0.00 |
| *AT4G36990* | *HSF4* | 0.00 | 0.00 | | -0.69 |
| *AT4G18880* | *HSF A4A* | 0.00 | 0.00 | | -0.60 |
| *AT5G62020* | *HSFB2A* | 0.00 | 0.00 | | -0.41 |
| *AT5G59720* | *HSP18.2* | 0.00 | 0.00 | | 0.44 |
| *AT3G23990* | *HSP60* | 0.00 | 0.00 | | 0.41 |
| *AT3G12580* | *HSP70* | 0.00 | 0.40 | | -0.37 |
| *AT5G52640* | *HSP90.1* | 0.00 | 0.00 | | -0.53 |
| *AT1G72970* | *HTH* | 0.00 | -0.56 | | 0.00 |
| *AT1G74520* | *HVA22A* | 0.00 | 0.00 | | -0.31 |
| *AT5G14200* | *IMD1* | 0.00 | -0.44 | | 0.00 |
| *AT1G31180* | *IMD3* | 0.00 | 0.00 | | 0.36 |
| *AT1G80920* | *J8* | 0.00 | 0.00 | | -0.53 |
| *AT4G36040* | *J11* | 0.00 | 0.53 | | -0.52 |
| *AT5G03260* | *LAC11* | 0.00 | 0.00 | | 0.55 |
| *AT4G15093* | *LigB* | 0.00 | 0.00 | | -0.66 |
| *AT1G75690* | *LQY1* | 0.00 | 0.00 | | 0.37 |
| *AT5G52310* | *LTI78/RD29A* | 0.00 | 0.00 | | -1.05 |
| *AT1G09970* | *LRR XI-23* | 0.00 | 0.00 | | -0.48 |
| *AT5G59320* | *LTP3* | -0.72 | -0.47 | | -0.98 |
| *AT5G59310* | *LTP4* | -0.97 | -0.43 | | -1.35 |
| *AT5G23010* | *MAM1* | 0.00 | -0.42 | | -0.54 |
| *AT1G63940* | *MDAR6* | 0.00 | 0.00 | | 0.55 |
| *AT2G22240* | *MIPS2* | 0.00 | 0.00 | | -0.79 |
| *AT1G70890* | *MLP43* | 0.00 | -0.32 | | -0.48 |
| *AT1G24020* | *MLP423* | 0.00 | -1.36 | | 0.00 |
| *AT3G45640* | *MPK3* | 0.00 | 0.00 | | -0.67 |
| *AT1G65290* | *mtACP2* | 0.00 | 0.00 | | 0.31 |
| *AT4G37910* | *mtHsc70-1* | 0.00 | 0.00 | | 0.35 |
| *AT5G67300* | *MYBR1* | 0.00 | 0.00 | | -0.44 |
| *AT2G47190* | *MYB2* | 0.00 | 1.01 | | 0.00 |
| *AT1G66230* | *MYB20* | 0.00 | 0.00 | | 0.51 |
| *AT5G07690* | *MYB29* | 0.00 | -0.75 | | 0.00 |
| *AT1G18710* | *MYB47* | 0.00 | 0.74 | | 0.00 |
| *AT4G34990* | *MYB32* | 0.00 | 0.00 | | -0.63 |
| *AT1G18570* | *MYB51* | 0.00 | 0.00 | | -0.57 |
| *AT1G71030* | *MYBL2* | 0.00 | 0.30 | | 0.00 |
| *AT2G46810* | *MYC70* | 0.00 | 0.00 | | 0.43 |
| *AT4G04840* | *MSRB6* | 0.00 | 0.00 | | 0.41 |
| *AT3G59970* | *MTHFR1* | 0.00 | -0.48 | | 0.00 |
| *AT2G44160* | *MTHFR2* | 0.00 | -0.33 | | 0.00 |
| *AT1G56010* | *NAC1* | 0.00 | 0.00 | | -1.14 |
| *AT5G39610* | *NAC6* | 0.00 | 0.48 | | -0.63 |
| *AT1G34180* | *NAC016* | 0.00 | 0.00 | | -0.75 |
| *AT1G52890* | *NAC019* | 0.00 | 0.80 | | 0.00 |
| *AT4G19170* | *NCED4* | 0.00 | 0.45 | | -0.42 |
| *AT5G63310* | *NDPK2* | 0.00 | 0.00 | | 0.80 |
| *AT2G15620* | *NIR1* | 0.00 | 0.00 | | 0.34 |
| *AT4G12720* | *NUDT7* | 0.00 | -0.73 | | -0.75 |
| *AT1G76680* | *OPR1* | 0.00 | 0.00 | | -0.44 |
| *AT2G19810* | *OZF1* | 0.00 | 0.00 | | -0.77 |
| *AT3G26830* | *PAD3* | 0.00 | 1.37 | | 0.00 |
| *AT2G43020* | *PAO2* | 0.00 | 0.00 | | -0.54 |
| *AT3G17790* | *PAP17* | 0.00 | 0.54 | | 0.00 |
| *AT5G60640* | *PDIL1-4* | 0.00 | 0.00 | | -0.40 |
| *AT1G17745* | *PGDH* | 0.00 | 0.00 | | -0.65 |
| *AT1G01620* | *PIP1C* | 0.00 | -0.68 | | 0.44 |
| *AT2G39010* | *PIP2E* | 0.00 | -0.38 | | 0.00 |
| *AT4G23400* | *PIP1;5* | 0.00 | -0.33 | | 0.00 |
| *AT2G16850* | *PIP2;8* | 0.00 | 0.00 | | 0.67 |
| *AT2G22170* | *PLAT2* | 0.00 | -1.20 | | 0.00 |
| *AT5G61640* | *PMSR1* | 0.00 | 0.00 | | -0.56 |
| *AT5G07460* | *PMSR2* | 0.38 | 0.00 | | 0.00 |
| *AT1G05850* | *POM1* | 0.00 | -0.40 | | 0.00 |
| *AT5G54190* | *PORA* | -1.17 | 0.00 | | 0.00 |
| *AT4G27440* | *PORB* | 0.00 | -0.31 | | 0.00 |
| *AT1G03630* | *PORC* | 0.00 | 0.00 | | 0.32 |
| *AT3G26060* | *PRXQ* | 0.00 | -0.34 | | 0.37 |
| *AT4G21960* | *PRXR1* | 0.00 | 0.00 | | -0.59 |
| *AT3G52150* | *PSRP2* | 0.00 | 0.00 | | 0.57 |
| *AT4G20960* | *PyrD* | 0.00 | -0.33 | | 0.00 |
| *AT5G66400* | *RAB18* | 0.00 | 0.00 | | -0.45 |
| *AT1G13260* | *RAV1* | 0.00 | 0.00 | | -0.41 |
| *AT5G38430* | *RBCS1B* | 0.00 | 0.00 | | 0.78 |
| *AT5G47910* | *RBOHD* | 0.00 | 0.00 | | -0.39 |
| *AT2G21620* | *RD2* | 0.00 | 0.00 | | -0.46 |
| *AT4G39090* | *RD19* | 0.00 | 0.00 | | -0.37 |
| *AT2G33380* | *RD20* | 0.00 | 0.00 | | -0.58 |
| *AT1G47128* | *RD21A* | 0.00 | 0.00 | | -0.38 |
| *AT2G37180* | *RD28* | 0.00 | 0.00 | | 0.35 |
| *AT3G46620* | *RDUF1* | 0.00 | 0.00 | | -0.40 |
| *AT5G59550* | *RDUF2* | 0.00 | 0.00 | | -0.44 |
| *AT3G62030* | *ROC4* | 0.00 | -0.33 | | 0.70 |
| *AT1G69270* | *RPK1* | 0.00 | 0.00 | | -0.45 |
| *AT5G14070* | *ROXY2* | 0.00 | 0.00 | | 1.54 |
| *AT4G15690* | *ROXY12* | -0.58 | 0.00 | | 1.47 |
| *AT4G15680* | *ROXY13* | -0.51 | 0.00 | | 2.23 |
| *AT4G15660* | *ROXY15* | -0.56 | 0.00 | | 0.00 |
| *AT3G62930* | *ROXY17* | -0.42 | 0.00 | | 1.44 |
| *AT2G29350* | *SAG13* | 0.00 | 0.63 | | -1.25 |
| *AT4G12040* | *SAP7* | 0.00 | 0.00 | | -0.56 |
| *AT2G26740* | *SEH* | -0.88 | -2.06 | | -1.12 |
| *AT2G21970* | *SEP2* | 0.00 | 0.00 | | -0.39 |
| *AT4G12420* | *SKU5* | 0.00 | -0.71 | | 0.38 |
| *AT5G08590* | *SNRK2.1* | 0.00 | 0.00 | | -0.32 |
| *AT1G78290* | *SNRK2-8* | 0.00 | 0.00 | | -0.45 |
| *AT5G24160* | *SQE6* | 0.00 | 0.59 | | 0.00 |
| *AT1G27730* | *STZ* | 0.00 | 0.00 | | -0.77 |
| *AT5G55220* | *TIG1* | 0.00 | 0.00 | | 0.45 |
| *AT4G09010* | *TL29* | 0.00 | 0.00 | | 0.48 |
| *AT3G27060* | *TSO2* | 0.00 | 0.00 | | 0.36 |
| *AT1G65980* | *TPX1* | 0.00 | 0.00 | | -0.35 |
| *AT3G06730* | *TRX Z* | 0.00 | 0.00 | | 0.34 |
| *AT1G45145* | *TRX5* | 0.00 | 0.00 | | -0.47 |
| *AT2G30250* | *WRKY25* | 0.00 | 0.00 | | -0.55 |
| *AT5G07100* | *WRKY26* | 0.00 | 0.68 | | 0.00 |
| *AT5G24110* | *WRKY30* | 0.00 | -0.95 | | 0.00 |
| *AT2G38470* | *WRKY33* | 0.00 | 0.00 | | -0.60 |
| *AT2G46400* | *WRKY46* | 0.00 | 0.00 | | -0.64 |
| *AT3G56400* | *WRKY70* | 0.00 | 0.00 | | -0.40 |
| *AT5G65730* | *XTH6* | 0.00 | -0.46 | | -0.31 |
| *AT5G04340* | *ZAT6* | 0.00 | 0.00 | | -0.94 |
| *AT3G19580* | *ZF2* | 0.00 | 0.00 | | -0.61 |
| *AT1G03410* | *2A6* | -0.59 | -0.45 | | -0.60 |
| *AT5G06290* | *2-Cys Prx B* | 0.00 | 0.00 | | 0.52 |
| *AT1G02360* | *AT1G02360* | 0.00 | -0.87 | | 0.00 |
| *AT1G04350* | *AT1G04350* | 0.00 | 0.00 | | -0.42 |
| *AT1G06640* | *AT1G06640* | 0.00 | -0.36 | | 0.42 |
| *AT1G13340* | *AT1G13340* | 0.00 | 0.61 | | 0.00 |
| *AT1G14810* | *AT1G14810* | 0.00 | 0.00 | | -0.33 |
| *AT1G21400* | *AT1G21400* | 0.00 | 0.00 | | -0.72 |
| *AT1G22190* | *AT1G22190* | 0.00 | 0.00 | | -2.12 |
| *AT1G26390* | *AT1G26390* | 0.00 | 0.86 | | -1.51 |
| *AT1G60730* | *AT1G60730* | 0.00 | 0.00 | | -0.50 |
| *AT1G64710* | *AT1G64710* | 0.00 | 0.00 | | -0.35 |
| *AT1G67360* | *AT1G67360* | 0.00 | 0.00 | | -0.92 |
| *AT1G69450* | *AT1G69450* | 0.00 | 0.00 | | 0.32 |
| *AT1G74470* | *AT1G74470* | 0.00 | 0.00 | | 2.09 |
| *AT1G77930* | *AT1G77930* | 0.00 | 0.00 | | -0.36 |
| *AT2G15960* | *AT2G15960* | 0.00 | 0.00 | | -0.41 |
| *AT2G17880* | *AT2G17880* | 0.00 | -0.54 | | 0.00 |
| *AT2G19310* | *AT2G19310* | 0.00 | 0.00 | | -0.37 |
| *AT2G20560* | *AT2G20560* | 0.00 | 0.53 | | 0.00 |
| *AT2G21860* | *AT2G21860* | -0.44 | 0.00 | | 0.00 |
| *AT2G22360* | *AT2G22360* | 0.00 | 0.00 | | 0.35 |
| *AT2G34620* | *AT2G34620* | 0.00 | -0.71 | | 0.46 |
| *AT2G47710* | *AT2G47710* | 0.00 | 0.00 | | -0.34 |
| *AT2G47880* | *AT2G47880* | 0.00 | 0.00 | | -0.64 |
| *AT3G09580* | *AT3G09580* | 0.00 | 0.00 | | 0.33 |
| *AT3G11630* | *AT3G11630* | 0.00 | -0.33 | | 0.55 |
| *AT3G19010* | *AT3G19010* | 0.00 | 0.00 | | -0.31 |
| *AT3G21460* | *AT3G21460* | 0.00 | 0.00 | | 1.41 |
| *AT3G28940* | *AT3G28940* | 0.00 | 0.00 | | -0.48 |
| *AT3G47650* | *AT3G47650* | 0.00 | 0.00 | | 0.34 |
| *AT3G52960* | *AT3G52960* | 0.00 | 0.00 | | 0.33 |
| *AT3G60290* | *AT3G60290* | 0.00 | 0.00 | | 0.31 |
| *AT3G62960* | *AT3G62960* | 0.00 | 0.00 | | -0.98 |
| *AT4G01700* | *AT4G01700* | 0.33 | 0.00 | | 0.00 |
| *AT4G10500* | *AT4G10500* | 0.00 | 0.58 | | 0.00 |
| *AT4G12290* | *AT4G12290* | 0.00 | 0.38 | | -1.04 |
| *AT4G15670* | *AT4G15670* | -0.62 | 0.00 | | 0.00 |
| *AT4G15700* | *AT4G15700* | -0.75 | 0.00 | | 1.58 |
| *AT4G20830* | *AT4G20830* | 0.00 | -0.55 | | 0.00 |
| *AT4G21580* | *AT4G21580* | 0.00 | 0.00 | | -0.38 |
| *AT4G25300* | *AT4G25300* | 0.00 | 0.00 | | -0.55 |
| *AT4G32208* | *AT4G32208* | 1.02 | 1.21 | | 1.06 |
| *AT4G33040* | *AT4G33040* | 0.00 | 0.39 | | -1.14 |
| *AT5G02160* | *AT5G02160* | 0.00 | 0.00 | | 0.32 |
| *AT5G05750* | *AT5G05750* | 0.00 | 0.00 | | -0.50 |
| *AT5G11930* | *AT5G11930* | 0.00 | 0.00 | | -0.95 |
| *AT5G13810* | *AT5G13810* | 0.00 | 0.00 | | -0.32 |
| *AT5G19875* | *AT5G19875* | 0.00 | 0.35 | | 0.00 |
| *AT5G22580* | *AT5G22580* | 0.00 | -1.35 | | -0.44 |
| *AT5G23240* | *AT5G23240* | 0.00 | 0.00 | | -1.35 |
| *AT5G24155* | *AT5G24155* | 0.00 | 0.59 | | 0.00 |
| *AT5G24890* | *AT5G24890* | 0.00 | 0.00 | | -0.32 |
| *AT5G35735* | *AT5G35735* | 0.00 | 0.00 | | -0.54 |
| *AT5G38710* | *AT5G38710* | 0.00 | 1.51 | | 0.00 |
| *AT5G42250* | *AT5G42250* | 0.00 | -0.52 | | -1.11 |
| *AT5G43440* | *AT5G43440* | -0.64 | 0.00 | | -0.99 |
| *AT5G43450* | *AT5G43450* | 0.37 | 0.00 | | 0.00 |
| *AT5G44400* | *AT5G44400* | 0.00 | -0.43 | | 0.00 |
| *AT5G44410* | *AT5G44410* | 0.00 | -0.31 | | -0.57 |
| *AT5G47590* | *AT5G47590* | 0.00 | 0.00 | | -1.17 |
| *AT5G51890* | *AT5G51890* | 0.00 | -0.51 | | 0.00 |
| *AT5G61820* | *AT5G61820* | 0.00 | 0.00 | | -0.46 |


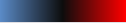


log10 fold change

+

-

**Fig. S4**
