# Supplementary material for: Transcriptomic analysis of poco1, a mitochondrial pentatricopeptide repeat protein mutant in Arabidopsis thaliana
Source: BMC Plant Biol. 2020 May 12;20:209. doi: 10.1186/s12870-020-02418-z (PMC7216612; doi:10.1186/s12870-020-02418-z)
Supplement: Supplementary file 6 — Additional file 6: Figure S5. Fold change heat map of genes encoding transcription factors. Fold change expression of genes encoding transcription factors is visualized by a heat map. The expression of different classes of transcription factors is affected in poco1. Fold changes (log10) were used for representing in the heat map. Red and blue represent up- and down-regulated transcripts respectively. Black represents that fold changes either ≥2 or ≤ − 2 with an FDR < 0.05 were not detected. TFs, transcription factors. [file 12870_2020_2418_MOESM6_ESM.ppt]

## Slide 1
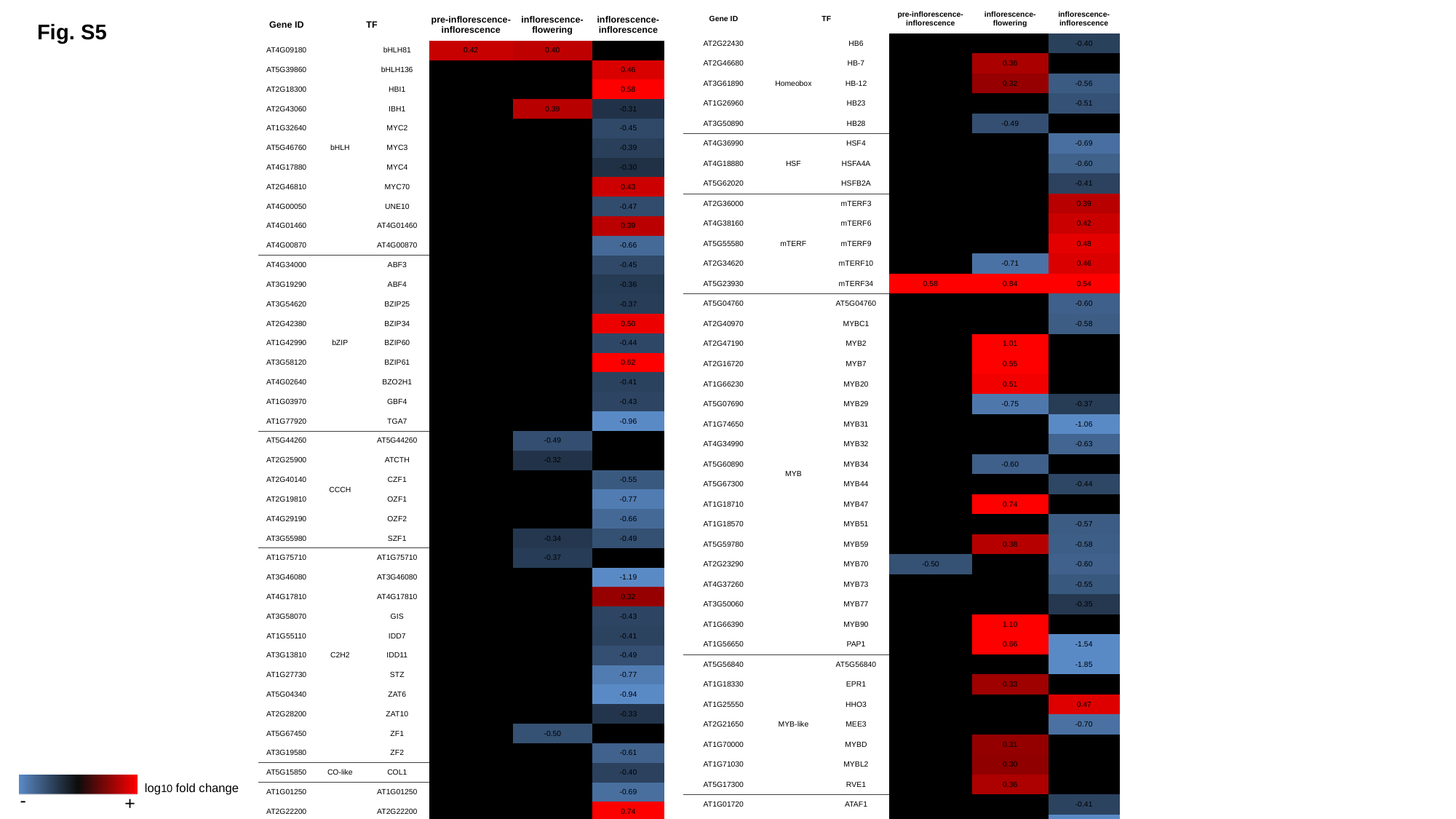

| Gene ID | TF | | pre-inflorescence-inflorescence | inflorescence-flowering | inflorescence-inflorescence |
| --- | --- | --- | --- | --- | --- |
| AT2G22430 | Homeobox | HB6 | 0.00 | 0.00 | -0.40 |
| AT2G46680 | | HB-7 | 0.00 | 0.36 | 0.00 |
| AT3G61890 | | HB-12 | 0.00 | 0.32 | -0.56 |
| AT1G26960 | | HB23 | 0.00 | 0.00 | -0.51 |
| AT3G50890 | | HB28 | 0.00 | -0.49 | 0.00 |
| AT4G36990 | HSF | HSF4 | 0.00 | 0.00 | -0.69 |
| AT4G18880 | | HSFA4A | 0.00 | 0.00 | -0.60 |
| AT5G62020 | | HSFB2A | 0.00 | 0.00 | -0.41 |
| AT2G36000 | mTERF | mTERF3 | 0.00 | 0.00 | 0.39 |
| AT4G38160 | | mTERF6 | 0.00 | 0.00 | 0.42 |
| AT5G55580 | | mTERF9 | 0.00 | 0.00 | 0.48 |
| AT2G34620 | | mTERF10 | 0.00 | -0.71 | 0.46 |
| AT5G23930 | | mTERF34 | 0.58 | 0.84 | 0.54 |
| AT5G04760 | MYB | AT5G04760 | 0.00 | 0.00 | -0.60 |
| AT2G40970 | | MYBC1 | 0.00 | 0.00 | -0.58 |
| AT2G47190 | | MYB2 | 0.00 | 1.01 | 0.00 |
| AT2G16720 | | MYB7 | 0.00 | 0.55 | 0.00 |
| AT1G66230 | | MYB20 | 0.00 | 0.51 | 0.00 |
| AT5G07690 | | MYB29 | 0.00 | -0.75 | -0.37 |
| AT1G74650 | | MYB31 | 0.00 | 0.00 | -1.06 |
| AT4G34990 | | MYB32 | 0.00 | 0.00 | -0.63 |
| AT5G60890 | | MYB34 | 0.00 | -0.60 | 0.00 |
| AT5G67300 | | MYB44 | 0.00 | 0.00 | -0.44 |
| AT1G18710 | | MYB47 | 0.00 | 0.74 | 0.00 |
| AT1G18570 | | MYB51 | 0.00 | 0.00 | -0.57 |
| AT5G59780 | | MYB59 | 0.00 | 0.38 | -0.58 |
| AT2G23290 | | MYB70 | -0.50 | 0.00 | -0.60 |
| AT4G37260 | | MYB73 | 0.00 | 0.00 | -0.55 |
| AT3G50060 | | MYB77 | 0.00 | 0.00 | -0.35 |
| AT1G66390 | | MYB90 | 0.00 | 1.10 | 0.00 |
| AT1G56650 | | PAP1 | 0.00 | 0.66 | -1.54 |
| AT5G56840 | MYB-like | AT5G56840 | 0.00 | 0.00 | -1.85 |
| AT1G18330 | | EPR1 | 0.00 | 0.33 | 0.00 |
| AT1G25550 | | HHO3 | 0.00 | 0.00 | 0.47 |
| AT2G21650 | | MEE3 | 0.00 | 0.00 | -0.70 |
| AT1G70000 | | MYBD | 0.00 | 0.31 | 0.00 |
| AT1G71030 | | MYBL2 | 0.00 | 0.30 | 0.00 |
| AT5G17300 | | RVE1 | 0.00 | 0.36 | 0.00 |
| AT1G01720 | NAC | ATAF1 | 0.00 | 0.00 | -0.41 |
| AT1G56010 | | NAC1 | 0.00 | 0.00 | -1.14 |
| AT3G15510 | | NAC2 | 0.00 | 0.00 | -0.95 |
| AT3G29035 | | NAC3 | 0.00 | 0.72 | -0.96 |
| AT5G39610 | | NAC6 | 0.00 | 0.48 | -0.63 |
| AT1G34180 | | NAC016 | 0.00 | 0.00 | -0.75 |
| AT1G69490 | | NAC018 | 0.00 | 0.32 | -0.67 |
| AT1G52890 | | NAC019 | 0.00 | 0.80 | 0.00 |
| AT1G69490 | | NAC029 | 0.00 | 0.33 | -0.64 |
| AT1G77450 | | NAC032 | 0.00 | 0.00 | -0.54 |
| AT2G17040 | | NAC036 | 0.00 | 0.00 | -0.55 |
| AT3G04060 | | NAC046 | 0.00 | 0.74 | 0.00 |
| AT5G13180 | | NAC083 | 0.00 | 0.38 | 0.00 |
| AT5G22290 | | NAC089 | 0.00 | 0.00 | -0.42 |
| AT1G52880 | | NAM | 0.00 | 0.32 | -0.67 |
| AT3G05690 | CCAAT | NF-YA2 | 0.00 | 0.34 | 0.00 |
| AT2G34720 | | NF-YA4 | 0.00 | 0.00 | -0.58 |
| AT5G06510 | | NF-YA10 | 0.00 | 0.00 | -0.94 |
| AT5G47640 | | NF-YB2 | 0.00 | 0.00 | -0.58 |
| AT1G52200 | PLATZ | AT1G52200 | 0.00 | 0.47 | 0.00 |
| AT1G21000 | | AT1G21000 | 0.00 | 0.00 | -0.70 |
| AT1G32700 | | AT1G32700 | 0.00 | 0.00 | -0.44 |
| AT1G76350 | RWP-RK | AT1G76350 | 0.00 | 0.00 | -0.53 |
| AT2G43500 | | AT2G43500 | 0.00 | 0.31 | -0.68 |
| AT1G25560 | RAV | TEM1 | 0.00 | 0.00 | -0.37 |
| AT5G24120 | Sigma70-like | SIGE | 0.00 | 0.00 | -0.50 |
| AT5G23280 | TCP | AT5G23280 | 0.00 | 0.00 | -0.38 |
| AT5G08330 | | TCP11 | 0.00 | -0.45 | 0.00 |
| AT5G56270 | WRKY | WRKY2 | 0.00 | 0.00 | -0.45 |
| AT2G24570 | | WRKY17 | 0.00 | 0.00 | 0.49 |
| AT2G23320 | | WRKY15 | 0.00 | -0.39 | -0.45 |
| AT2G30250 | | WRKY25 | 0.00 | 0.00 | -0.55 |
| AT5G07100 | | WRKY26 | 0.00 | 0.68 | 0.00 |
| AT5G24110 | | WRKY30 | 0.00 | -0.95 | 0.00 |
| AT2G38470 | | WRKY33 | 0.00 | 0.00 | -0.60 |
| AT2G46400 | | WRKY46 | 0.00 | 0.00 | -0.64 |
| AT4G23810 | | WRKY53 | 0.00 | -0.44 | 0.00 |
| AT3G56400 | | WRKY70 | 0.00 | 0.00 | -0.40 |
| Gene ID | TF | | pre-inflorescence-inflorescence | inflorescence-flowering | inflorescence-inflorescence |
| --- | --- | --- | --- | --- | --- |
| AT4G09180 | bHLH | bHLH81 | 0.42 | 0.40 | 0.00 |
| AT5G39860 | | bHLH136 | 0.00 | 0.00 | 0.46 |
| AT2G18300 | | HBI1 | 0.00 | 0.00 | 0.58 |
| AT2G43060 | | IBH1 | 0.00 | 0.39 | -0.31 |
| AT1G32640 | | MYC2 | 0.00 | 0.00 | -0.45 |
| AT5G46760 | | MYC3 | 0.00 | 0.00 | -0.39 |
| AT4G17880 | | MYC4 | 0.00 | 0.00 | -0.30 |
| AT2G46810 | | MYC70 | 0.00 | 0.00 | 0.43 |
| AT4G00050 | | UNE10 | 0.00 | 0.00 | -0.47 |
| AT4G01460 | | AT4G01460 | 0.00 | 0.00 | 0.39 |
| AT4G00870 | | AT4G00870 | 0.00 | 0.00 | -0.66 |
| AT4G34000 | bZIP | ABF3 | 0.00 | 0.00 | -0.45 |
| AT3G19290 | | ABF4 | 0.00 | 0.00 | -0.36 |
| AT3G54620 | | BZIP25 | 0.00 | 0.00 | -0.37 |
| AT2G42380 | | BZIP34 | 0.00 | 0.00 | 0.50 |
| AT1G42990 | | BZIP60 | 0.00 | 0.00 | -0.44 |
| AT3G58120 | | BZIP61 | 0.00 | 0.00 | 0.62 |
| AT4G02640 | | BZO2H1 | 0.00 | 0.00 | -0.41 |
| AT1G03970 | | GBF4 | 0.00 | 0.00 | -0.43 |
| AT1G77920 | | TGA7 | 0.00 | 0.00 | -0.96 |
| AT5G44260 | CCCH | AT5G44260 | 0.00 | -0.49 | 0.00 |
| AT2G25900 | | ATCTH | 0.00 | -0.32 | 0.00 |
| AT2G40140 | | CZF1 | 0.00 | 0.00 | -0.55 |
| AT2G19810 | | OZF1 | 0.00 | 0.00 | -0.77 |
| AT4G29190 | | OZF2 | 0.00 | 0.00 | -0.66 |
| AT3G55980 | | SZF1 | 0.00 | -0.34 | -0.49 |
| AT1G75710 | C2H2 | AT1G75710 | 0.00 | -0.37 | 0.00 |
| AT3G46080 | | AT3G46080 | 0.00 | 0.00 | -1.19 |
| AT4G17810 | | AT4G17810 | 0.00 | 0.00 | 0.32 |
| AT3G58070 | | GIS | 0.00 | 0.00 | -0.43 |
| AT1G55110 | | IDD7 | 0.00 | 0.00 | -0.41 |
| AT3G13810 | | IDD11 | 0.00 | 0.00 | -0.49 |
| AT1G27730 | | STZ | 0.00 | 0.00 | -0.77 |
| AT5G04340 | | ZAT6 | 0.00 | 0.00 | -0.94 |
| AT2G28200 | | ZAT10 | 0.00 | 0.00 | -0.33 |
| AT5G67450 | | ZF1 | 0.00 | -0.50 | 0.00 |
| AT3G19580 | | ZF2 | 0.00 | 0.00 | -0.61 |
| AT5G15850 | CO-like | COL1 | 0.00 | 0.00 | -0.40 |
| AT1G01250 | ERF | AT1G01250 | 0.00 | 0.00 | -0.69 |
| AT2G22200 | | AT2G22200 | 0.00 | 0.00 | 0.74 |
| AT4G16750 | | AT4G16750 | 0.00 | 0.00 | -0.50 |
| AT4G32800 | | AT4G32800 | 0.00 | -0.47 | 0.00 |
| AT5G07580 | | AT5G07580 | 0.00 | 0.00 | -0.57 |
| AT5G51190 | | AT5G51190 | 0.00 | -0.77 | -0.87 |
| AT5G61590 | | AT5G61590 | -0.33 | 0.00 | -0.45 |
| AT4G23750 | | CRF2 | 0.00 | 0.00 | 0.73 |
| AT3G61630 | | CRF6 | 0.00 | 0.35 | 0.00 |
| AT1G22985 | | CRF7 | 0.00 | 0.00 | -0.42 |
| AT1G71130 | | CRF8 | 0.00 | 0.00 | -0.34 |
| AT2G23340 | | DEAR3 | 0.00 | 0.00 | -0.34 |
| AT3G16770 | | EBP | 0.00 | 0.47 | 0.00 |
| AT5G47220 | | ERF2 | 0.00 | 0.00 | -0.49 |
| AT3G15210 | | ERF4 | 0.00 | 0.00 | -0.39 |
| AT5G47230 | | ERF5 | 0.00 | -0.44 | -0.58 |
| AT1G28370 | | ERF11 | 0.00 | 0.00 | -0.66 |
| AT4G17490 | | ERF6 | 0.00 | -0.93 | -0.86 |
| AT2G31230 | | ERF15 | 0.00 | 0.00 | -0.61 |
| AT5G61600 | | ERF104 | 0.00 | -0.48 | -0.90 |
| AT5G25190 | | ESE3 | 0.00 | -0.57 | 0.72 |
| AT1G06160 | | ORA59 | 0.00 | 0.56 | -0.58 |
| AT4G36900 | | RAP2.10 | 0.00 | 0.00 | -0.77 |
| AT1G53910 | | RAP2.12 | 0.00 | 0.00 | -0.34 |
| AT1G13260 | | RAV1 | 0.00 | 0.00 | -0.41 |
| AT5G11590 | | TINY2 | 0.00 | -0.56 | -0.76 |
| AT4G16141 | GATA | AT4G16141 | 0.00 | 0.00 | 0.62 |
| AT4G36240 | | GATA7 | 0.00 | -0.53 | 0.00 |
| AT1G07520 | GRAS | AT1G07520 | 0.00 | 0.00 | -0.45 |
| AT3G46600 | | AT3G46600 | 0.00 | 0.00 | -0.37 |
| AT4G17230 | | SCL13 | 0.00 | 0.00 | -0.55 |
| AT1G07530 | | SCL14 | 0.00 | 0.00 | -0.34 |
| AT1G20693 | HMG | HMGB2 | 0.00 | 0.41 | 0.00 |
| AT2G35940 | Homeobox | BLH1 | 0.00 | 0.00 | -0.36 |
| AT1G75410 | | BLH3 | 0.00 | 0.00 | -0.37 |
| AT1G19700 | | BEL10 | 0.00 | 0.00 | -0.36 |
| AT4G16780 | | HB-2 | 0.00 | 0.00 | -0.33 |
| AT5G47370 | | HB-4 | 0.00 | 0.31 | 0.00 |
Fig. S5
log10 fold change
-
+
