# Supplementary material for: Transcriptomic analysis of poco1, a mitochondrial pentatricopeptide repeat protein mutant in Arabidopsis thaliana
Source: BMC Plant Biol. 2020 May 12;20:209. doi: 10.1186/s12870-020-02418-z (PMC7216612; doi:10.1186/s12870-020-02418-z)
Supplement: Supplementary file 7 — Additional file 7: Figure S6. Alteration of expression of genes associated with cellular signaling and mitochondrial perturbation targets in poco1. Genes associated with cellular signaling and mitochondrial perturbation were found differentially regulated in poco1. Fold changes (log10) were used for representing in the heat map. Red and blue represent up- and down-regulated transcripts respectively. Black represents that fold changes either ≥2 or ≤ − 2 with an FDR < 0.05 were not detected. Fold changes are relative to wild-type. [file 12870_2020_2418_MOESM7_ESM.ppt]

## Slide 1
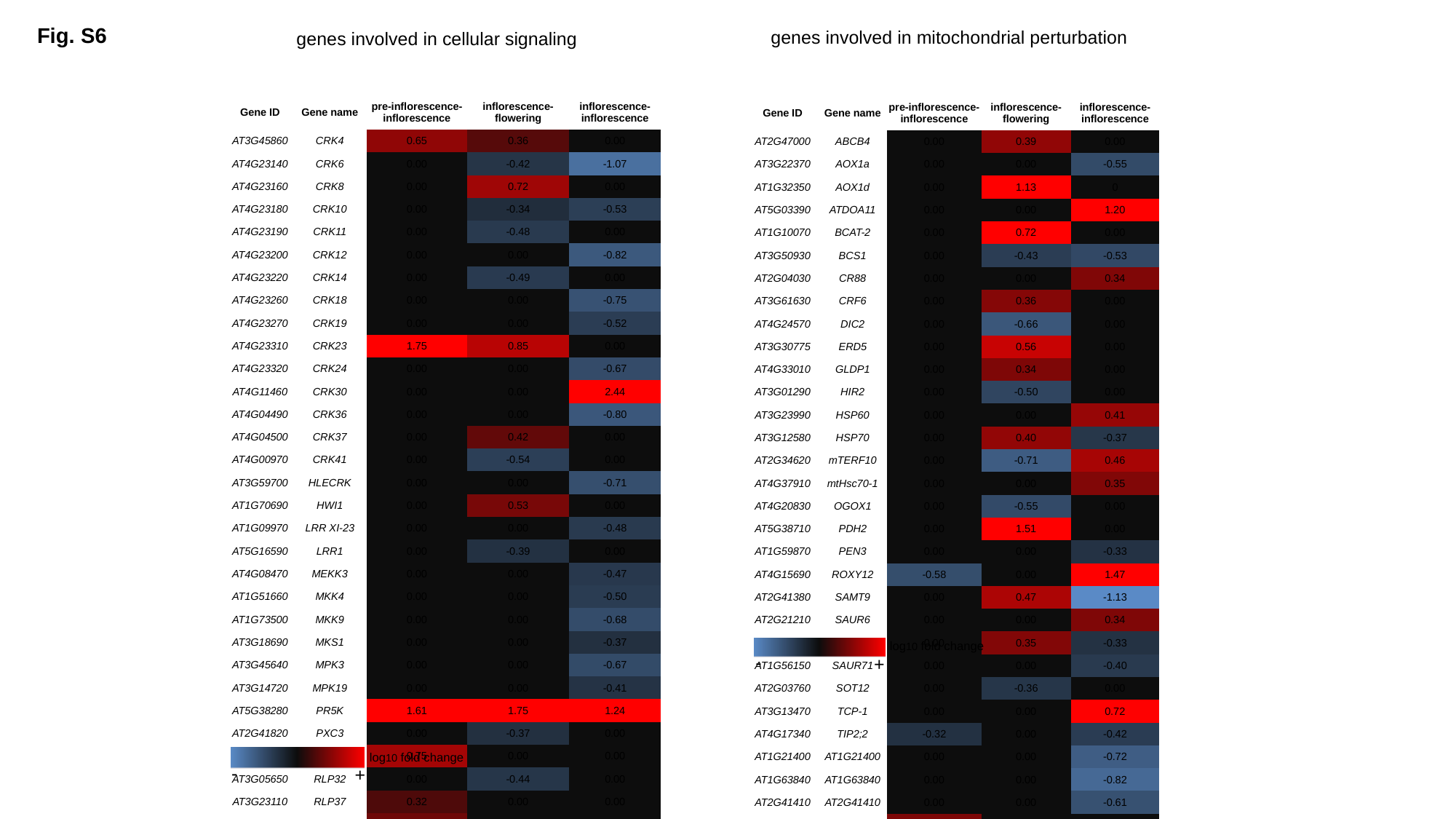

Fig. S6
genes involved in mitochondrial perturbation
genes involved in cellular signaling
| Gene ID | Gene name | pre-inflorescence-inflorescence | inflorescence-flowering | inflorescence-inflorescence |
| --- | --- | --- | --- | --- |
| AT3G45860 | CRK4 | 0.65 | 0.36 | 0.00 |
| AT4G23140 | CRK6 | 0.00 | -0.42 | -1.07 |
| AT4G23160 | CRK8 | 0.00 | 0.72 | 0.00 |
| AT4G23180 | CRK10 | 0.00 | -0.34 | -0.53 |
| AT4G23190 | CRK11 | 0.00 | -0.48 | 0.00 |
| AT4G23200 | CRK12 | 0.00 | 0.00 | -0.82 |
| AT4G23220 | CRK14 | 0.00 | -0.49 | 0.00 |
| AT4G23260 | CRK18 | 0.00 | 0.00 | -0.75 |
| AT4G23270 | CRK19 | 0.00 | 0.00 | -0.52 |
| AT4G23310 | CRK23 | 1.75 | 0.85 | 0.00 |
| AT4G23320 | CRK24 | 0.00 | 0.00 | -0.67 |
| AT4G11460 | CRK30 | 0.00 | 0.00 | 2.44 |
| AT4G04490 | CRK36 | 0.00 | 0.00 | -0.80 |
| AT4G04500 | CRK37 | 0.00 | 0.42 | 0.00 |
| AT4G00970 | CRK41 | 0.00 | -0.54 | 0.00 |
| AT3G59700 | HLECRK | 0.00 | 0.00 | -0.71 |
| AT1G70690 | HWI1 | 0.00 | 0.53 | 0.00 |
| AT1G09970 | LRR XI-23 | 0.00 | 0.00 | -0.48 |
| AT5G16590 | LRR1 | 0.00 | -0.39 | 0.00 |
| AT4G08470 | MEKK3 | 0.00 | 0.00 | -0.47 |
| AT1G51660 | MKK4 | 0.00 | 0.00 | -0.50 |
| AT1G73500 | MKK9 | 0.00 | 0.00 | -0.68 |
| AT3G18690 | MKS1 | 0.00 | 0.00 | -0.37 |
| AT3G45640 | MPK3 | 0.00 | 0.00 | -0.67 |
| AT3G14720 | MPK19 | 0.00 | 0.00 | -0.41 |
| AT5G38280 | PR5K | 1.61 | 1.75 | 1.24 |
| AT2G41820 | PXC3 | 0.00 | -0.37 | 0.00 |
| AT2G32680 | RLP23 | 0.75 | 0.00 | 0.00 |
| AT3G05650 | RLP32 | 0.00 | -0.44 | 0.00 |
| AT3G23110 | RLP37 | 0.32 | 0.00 | 0.00 |
| AT3G23120 | RLP38 | 0.48 | 0.00 | 0.00 |
| AT3G28890 | RLP43 | 0.00 | 0.00 | -0.85 |
| AT4G13920 | RLP50 | 0.00 | -0.86 | -1.36 |
| AT1G69270 | RPK1 | 0.00 | 0.00 | -0.45 |
| AT1G12460 | AT1G12460 | 0.00 | -0.56 | 0.00 |
| AT1G29720 | AT1G29720 | 0.00 | -0.35 | 0.00 |
| AT2G02780 | AT2G02780 | 0.00 | -0.38 | 0.00 |
| AT2G32140 | AT2G32140 | 0.00 | 0.00 | -1.00 |
| AT3G22060 | AT3G22060 | 0.34 | 0.00 | 0.00 |
| AT3G47090 | AT3G47090 | 0.00 | 0.00 | -0.42 |
| AT4G00300 | AT4G00300 | 0.00 | -0.45 | 0.00 |
| AT4G11521 | AT4G11521 | -0.38 | -0.51 | -0.47 |
| AT5G39020 | AT5G39020 | 0.00 | -0.31 | -0.81 |
| AT5G48540 | AT5G48540 | 0.00 | -0.53 | 0.00 |
| AT5G59670 | AT5G59670 | 0.68 | 0.00 | 0.00 |
| AT5G63410 | AT5G63410 | 0.00 | 0.00 | -0.48 |
| Gene ID | Gene name | pre-inflorescence-inflorescence | inflorescence-flowering | inflorescence-inflorescence |
| --- | --- | --- | --- | --- |
| AT2G47000 | ABCB4 | 0.00 | 0.39 | 0.00 |
| AT3G22370 | AOX1a | 0.00 | 0.00 | -0.55 |
| AT1G32350 | AOX1d | 0.00 | 1.13 | 0 |
| AT5G03390 | ATDOA11 | 0.00 | 0.00 | 1.20 |
| AT1G10070 | BCAT-2 | 0.00 | 0.72 | 0.00 |
| AT3G50930 | BCS1 | 0.00 | -0.43 | -0.53 |
| AT2G04030 | CR88 | 0.00 | 0.00 | 0.34 |
| AT3G61630 | CRF6 | 0.00 | 0.36 | 0.00 |
| AT4G24570 | DIC2 | 0.00 | -0.66 | 0.00 |
| AT3G30775 | ERD5 | 0.00 | 0.56 | 0.00 |
| AT4G33010 | GLDP1 | 0.00 | 0.34 | 0.00 |
| AT3G01290 | HIR2 | 0.00 | -0.50 | 0.00 |
| AT3G23990 | HSP60 | 0.00 | 0.00 | 0.41 |
| AT3G12580 | HSP70 | 0.00 | 0.40 | -0.37 |
| AT2G34620 | mTERF10 | 0.00 | -0.71 | 0.46 |
| AT4G37910 | mtHsc70-1 | 0.00 | 0.00 | 0.35 |
| AT4G20830 | OGOX1 | 0.00 | -0.55 | 0.00 |
| AT5G38710 | PDH2 | 0.00 | 1.51 | 0.00 |
| AT1G59870 | PEN3 | 0.00 | 0.00 | -0.33 |
| AT4G15690 | ROXY12 | -0.58 | 0.00 | 1.47 |
| AT2G41380 | SAMT9 | 0.00 | 0.47 | -1.13 |
| AT2G21210 | SAUR6 | 0.00 | 0.00 | 0.34 |
| AT3G60690 | SAUR59 | 0.00 | 0.35 | -0.33 |
| AT1G56150 | SAUR71 | 0.00 | 0.00 | -0.40 |
| AT2G03760 | SOT12 | 0.00 | -0.36 | 0.00 |
| AT3G13470 | TCP-1 | 0.00 | 0.00 | 0.72 |
| AT4G17340 | TIP2;2 | -0.32 | 0.00 | -0.42 |
| AT1G21400 | AT1G21400 | 0.00 | 0.00 | -0.72 |
| AT1G63840 | AT1G63840 | 0.00 | 0.00 | -0.82 |
| AT2G41410 | AT2G41410 | 0.00 | 0.00 | -0.61 |
| AT4G01700 | AT4G01700 | 0.33 | 0.00 | 0.00 |
| AT4G36500 | AT4G36500 | 0.00 | 0.00 | -0.47 |
| AT5G43150 | AT5G43150 | 0.00 | 0.00 | -0.85 |
| AT5G43450 | AT5G43450 | 0.37 | 0.00 | 0.00 |
| AT5G52970 | AT5G52970 | 0.00 | 0.00 | 0.47 |
| AT5G53880 | AT5G53880 | 0.00 | -0.39 | -0.38 |
| AT5G54130 | AT5G54130 | 0.00 | 0.38 | 0.00 |
log10 fold change
-
+
log10 fold change
-
+
