# Supplementary material for: Transcriptomic analysis of poco1, a mitochondrial pentatricopeptide repeat protein mutant in Arabidopsis thaliana
Source: BMC Plant Biol. 2020 May 12;20:209. doi: 10.1186/s12870-020-02418-z (PMC7216612; doi:10.1186/s12870-020-02418-z)
Supplement: Supplementary file 8 — Additional file 8: Figure S7. Expression alteration in redox-related genes and genes associated with stomatal function. Genes associated with the redox status and stomatal function were found to have differential regulation in poco1. Fold changes (log10) were used for representing in heat maps. Red and blue represent up- and down-regulated transcripts respectively. Black represents that fold changes either ≥2 or ≤ − 2 with an FDR < 0.05 were not detected. Fold changes are relative to wild-type. [file 12870_2020_2418_MOESM8_ESM.ppt]

## Slide 1
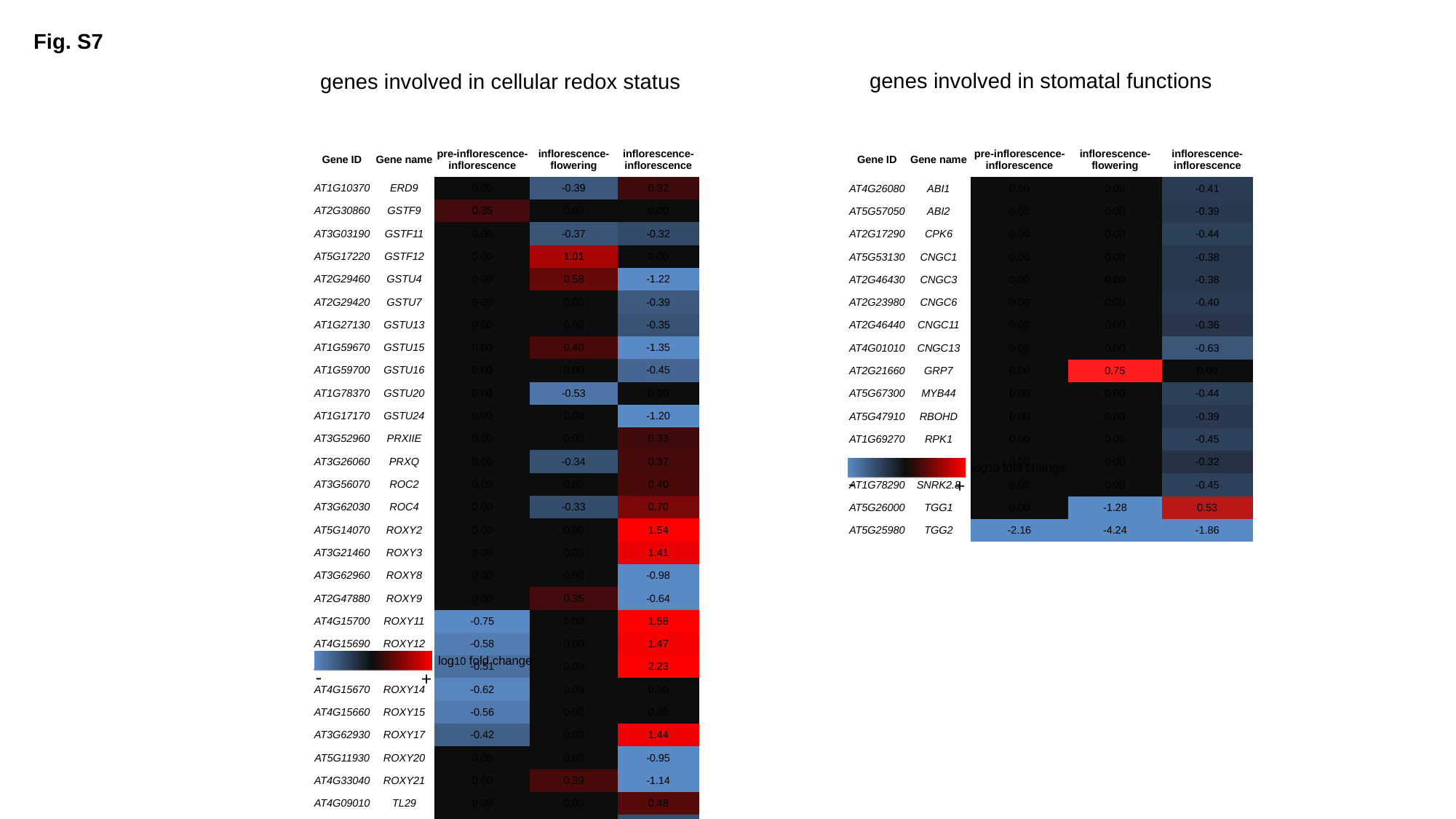

Fig. S7
genes involved in stomatal functions
genes involved in cellular redox status
| Gene ID | Gene name | pre-inflorescence-inflorescence | inflorescence-flowering | inflorescence-inflorescence |
| --- | --- | --- | --- | --- |
| AT1G10370 | ERD9 | 0.00 | -0.39 | 0.32 |
| AT2G30860 | GSTF9 | 0.35 | 0.00 | 0.00 |
| AT3G03190 | GSTF11 | 0.00 | -0.37 | -0.32 |
| AT5G17220 | GSTF12 | 0.00 | 1.01 | 0.00 |
| AT2G29460 | GSTU4 | 0.00 | 0.58 | -1.22 |
| AT2G29420 | GSTU7 | 0.00 | 0.00 | -0.39 |
| AT1G27130 | GSTU13 | 0.00 | 0.00 | -0.35 |
| AT1G59670 | GSTU15 | 0.00 | 0.40 | -1.35 |
| AT1G59700 | GSTU16 | 0.00 | 0.00 | -0.45 |
| AT1G78370 | GSTU20 | 0.00 | -0.53 | 0.00 |
| AT1G17170 | GSTU24 | 0.00 | 0.00 | -1.20 |
| AT3G52960 | PRXIIE | 0.00 | 0.00 | 0.33 |
| AT3G26060 | PRXQ | 0.00 | -0.34 | 0.37 |
| AT3G56070 | ROC2 | 0.00 | 0.00 | 0.40 |
| AT3G62030 | ROC4 | 0.00 | -0.33 | 0.70 |
| AT5G14070 | ROXY2 | 0.00 | 0.00 | 1.54 |
| AT3G21460 | ROXY3 | 0.00 | 0.00 | 1.41 |
| AT3G62960 | ROXY8 | 0.00 | 0.00 | -0.98 |
| AT2G47880 | ROXY9 | 0.00 | 0.35 | -0.64 |
| AT4G15700 | ROXY11 | -0.75 | 0.00 | 1.58 |
| AT4G15690 | ROXY12 | -0.58 | 0.00 | 1.47 |
| AT4G15680 | ROXY13 | -0.51 | 0.00 | 2.23 |
| AT4G15670 | ROXY14 | -0.62 | 0.00 | 0.00 |
| AT4G15660 | ROXY15 | -0.56 | 0.00 | 0.00 |
| AT3G62930 | ROXY17 | -0.42 | 0.00 | 1.44 |
| AT5G11930 | ROXY20 | 0.00 | 0.00 | -0.95 |
| AT4G33040 | ROXY21 | 0.00 | 0.39 | -1.14 |
| AT4G09010 | TL29 | 0.00 | 0.00 | 0.48 |
| AT1G65980 | TPX1 | 0.00 | 0.00 | -0.35 |
| AT3G06730 | TRX Z | 0.00 | 0.00 | 0.34 |
| AT1G45145 | TRX5 | 0.00 | 0.00 | -0.47 |
| AT3G11630 | 2CPA | 0.00 | -0.33 | 0.55 |
| AT5G06290 | 2CPB | 0.00 | 0.00 | 0.52 |
| AT5G13810 | AT5G13810 | 0.00 | 0.00 | -0.32 |
| Gene ID | Gene name | pre-inflorescence-inflorescence | inflorescence-flowering | inflorescence-inflorescence |
| --- | --- | --- | --- | --- |
| AT4G26080 | ABI1 | 0.00 | 0.00 | -0.41 |
| AT5G57050 | ABI2 | 0.00 | 0.00 | -0.39 |
| AT2G17290 | CPK6 | 0.00 | 0.00 | -0.44 |
| AT5G53130 | CNGC1 | 0.00 | 0.00 | -0.38 |
| AT2G46430 | CNGC3 | 0.00 | 0.00 | -0.38 |
| AT2G23980 | CNGC6 | 0.00 | 0.00 | -0.40 |
| AT2G46440 | CNGC11 | 0.00 | 0.00 | -0.36 |
| AT4G01010 | CNGC13 | 0.00 | 0.00 | -0.63 |
| AT2G21660 | GRP7 | 0.00 | 0.75 | 0.00 |
| AT5G67300 | MYB44 | 0.00 | 0.00 | -0.44 |
| AT5G47910 | RBOHD | 0.00 | 0.00 | -0.39 |
| AT1G69270 | RPK1 | 0.00 | 0.00 | -0.45 |
| AT5G08590 | SNRK2.1 | 0.00 | 0.00 | -0.32 |
| AT1G78290 | SNRK2.8 | 0.00 | 0.00 | -0.45 |
| AT5G26000 | TGG1 | 0.00 | -1.28 | 0.53 |
| AT5G25980 | TGG2 | -2.16 | -4.24 | -1.86 |
log10 fold change
-
+
log10 fold change
-
+
